# Broadening the functionality of a J-protein/Hsp70 molecular chaperone system

**DOI:** 10.1371/journal.pgen.1007084

**Published:** 2017-10-30

**Authors:** Brenda A. Schilke, Szymon J. Ciesielski, Thomas Ziegelhoffer, Erina Kamiya, Marco Tonelli, Woonghee Lee, Gabriel Cornilescu, Justin K. Hines, John L. Markley, Elizabeth A. Craig

**Affiliations:** 1 Department of Biochemistry, University of Wisconsin-Madison, Madison, Wisconsin, United States of America; 2 Department of Chemistry, Lafayette College, Easton, Pennsylvania, United States of America; 3 National Magnetic Resonance Facility at Madison, University of Wisconsin-Madison, Madison, Wisconsin, United States of America; University of Michigan, UNITED STATES

## Abstract

By binding to a multitude of polypeptide substrates, Hsp70-based molecular chaperone systems perform a range of cellular functions. All J-protein co-chaperones play the essential role, via action of their J-domains, of stimulating the ATPase activity of Hsp70, thereby stabilizing its interaction with substrate. In addition, J-proteins drive the functional diversity of Hsp70 chaperone systems through action of regions outside their J-domains. Targeting to specific locations within a cellular compartment and binding of specific substrates for delivery to Hsp70 have been identified as modes of J-protein specialization. To better understand J-protein specialization, we concentrated on *Saccharomyces cerevisiae SIS1*, which encodes an essential J-protein of the cytosol/nucleus. We selected suppressors that allowed cells lacking *SIS1* to form colonies. Substitutions changing single residues in Ydj1, a J-protein, which, like Sis1, partners with Hsp70 Ssa1, were isolated. These gain-of-function substitutions were located at the end of the J-domain, suggesting that suppression was connected to interaction with its partner Hsp70, rather than substrate binding or subcellular localization. Reasoning that, if *YDJ1* suppressors affect Ssa1 function, substitutions in Hsp70 itself might also be able to overcome the cellular requirement for Sis1, we carried out a selection for *SSA1* suppressor mutations. Suppressing substitutions were isolated that altered sites in Ssa1 affecting the cycle of substrate interaction. Together, our results point to a third, additional means by which J-proteins can drive Hsp70’s ability to function in a wide range of cellular processes—modulating the Hsp70-substrate interaction cycle.

## Author summary

Molecular chaperones are proteins that bind transiently with other proteins, facilitating their effective function by, for example, aiding their folding, degradation or interaction with other proteins. Hsp70-based chaperone systems are arguably the most versatile. Much of their ability to specialize is driven by obligate J-protein co-chaperones. Two ways in which J-proteins can drive Hsp70 specialization are known: by physical localizing to a particular site of action, recruiting Hsp70 to substrates present there; and by binding substrates directly, giving them a “leg up” to bind Hsp70. Using the genetic system of budding yeast, we found evidence for a third means by which J-proteins can drive Hsp70 versatility—tuning the cycle of Hsp70’s interaction with substrate proteins. As this is a step common to all Hsp70 systems, we suggest that our results are likely applicable to many Hsp70 systems.

## Introduction

Hsp70-based molecular chaperone machineries function in a wide range of cellular processes, including folding of nascent polypeptide chains as they emerge from ribosomes, driving protein translocation across membranes, preventing protein aggregation and facilitating biogenesis of Fe/S clusters [1, 2]. Regardless of their specific functional role in the cell, all Hsp70s use the same fundamental biochemical mechanism of action—cycles of interaction with substrate proteins driven by ATP binding and hydrolysis [3]. ATP hydrolysis, stimulated by interaction of both substrate and a J-protein co-chaperone, results in trapping of substrate. Nucleotide exchange factor drives release of nucleotide and, thus, substrate release [4]. Much of the functional versatility of Hsp70 is due to its interaction with an array of different J-protein co-chaperones [5]. Two means by which J-proteins drive this diversity have been well documented: the binding of substrate by a J-protein, thereby “delivering” it to Hsp70 and localization of a J-protein within a cellular compartment, thereby recruiting Hsp70 to the cellular site of particular substrates. These roles are performed by sequences distinct from their J-domain, which is responsible for stimulation of Hsp70’s ATPase activity.

**Fig 1:**
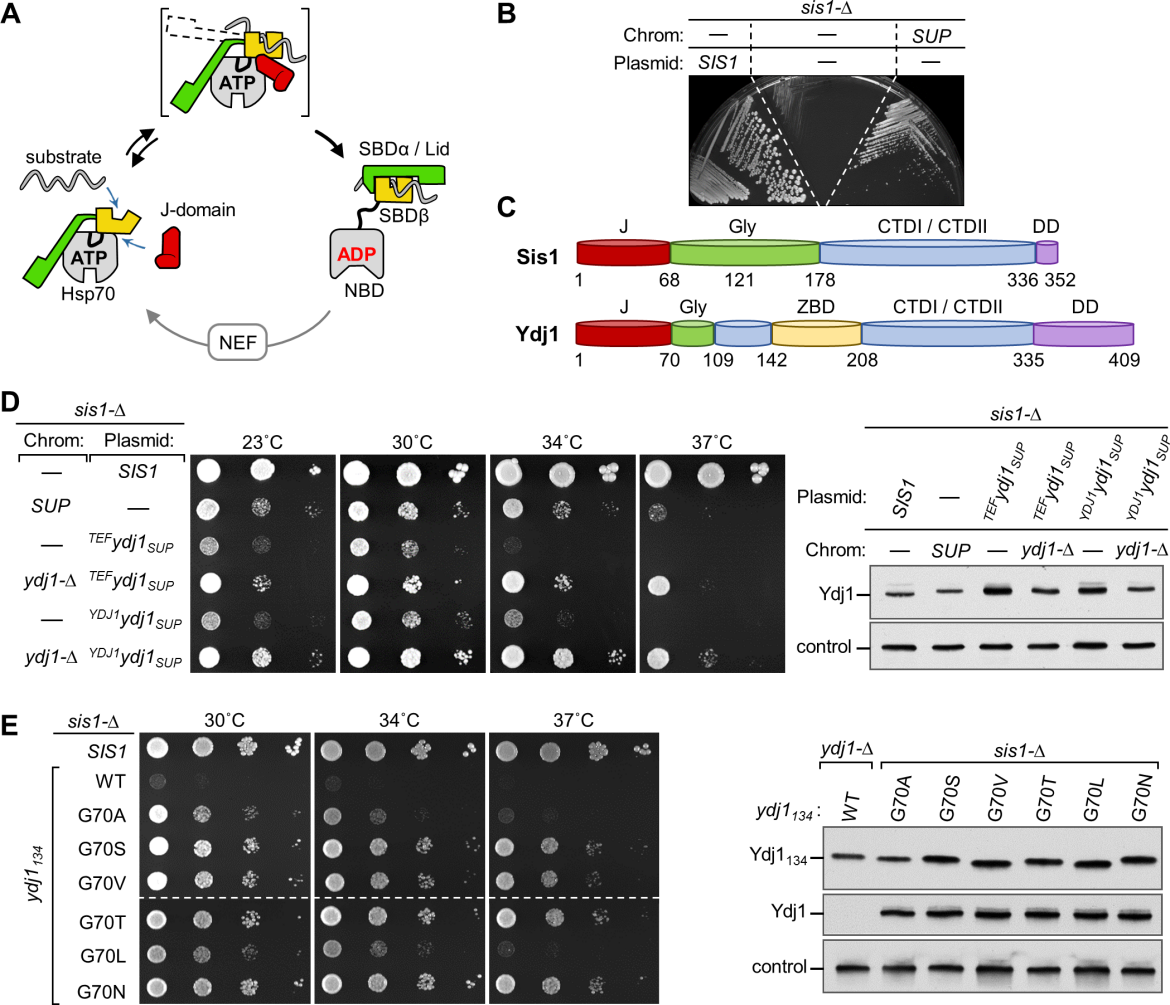
Isolation of a spontaneous *YDJ1* suppressor of *sis1-Δ*. **(A)** Overview of Hsp70 interaction with J-domain and substrate. When Hsp70 is bound to ATP (left), substrate has easy access to the cleft in the β-subdomain of SBD (SBDβ). This is often called the open-or docked-state, because the lid (SBDα), which traps substrate by covering the cleft when ADP is bound (right), is restrained through interaction with the NBD; the SBDβ and inter-domain linker are also docked on the NBD. In concert with substrate in the cleft, J-domain binding at the NBD-SBDβ interface stimulates hydrolysis of ATP to ADP. The resulting conformational changes cause the domains to disengage, forming the undocked/closed state and stabilizing substrate interaction. Bracket indicates dynamic transitions between predominant ATP and ADP conformations with dotted line indicating movement of the SBDα lid. Nucleotide release by nucleotide exchange factors (NEF) and rebinding of ATP completes the cycle (bottom). SBDα (green), SBDβ (yellow), NBD (gray), linker (black). (**B**) *sis1-Δ* cells carrying a plasmid with a wild type (WT) *SIS1* gene and *URA3* marker, plus the indicated additional plasmid having a *TRP1* marker, and/or having the genomic suppressor mutation were streaked on plates containing 5-FOA. Plasmid with *TRP1* marker: WT copy of *SIS1* (*SIS1*) or no insert (-); on the chromosome (Chrom): SUP indicates presence of spontaneous suppressor mutation. Plate was incubated for 3 days at 30°C. (**C**) Schematic representation of Sis1 and Ydj1 architecture. J, J-domain (red); Gly, Glycine-rich region (green); CTDI/CTDII, C-terminal domains I and II, each of which is a b-barrel (blue); ZBD, zinc binding domain (yellow); DD, dimerization domain (purple). Note: the Sis1 Gly-rich region is often divided into a G/F region (residues 69–121) and the G/M region (122–177), as the former is rich in phenylalanine, and the later in methionine, as well glycine. (**D**) (*left*) Ten-fold serial dilutions of *sis1-Δ* strains with no additional chromosomal mutation (-), a deletion of *YDJ1* (*ydj1-Δ*), or the suppressor mutation (SUP) were grown at indicated temperatures for 3 days. Strains also carried a *TRP1* plasmid: no insert (-) or the *YDJ1* gene cloned from the suppressor strain (*ydj1*_*SUP*_*)* under the control of its native promoter or the *TEF2* promoter (superscript *YDJ1* or *TEF*, respectively). (*right*) Cell lysates of strains were subjected to immunoblot analysis using antibodies specific for the Ydj1 J-domain or, as a control, Ssc1. (**E**) (*left*) Comparison of growth of a *sis1-Δ* strain with a plasmid carrying WT *SIS1* or, under control of the *TEF2* promoter, a truncated *YDJ1* encoding the N-terminal 134 residues (*YDJ1*_*134*_), either WT in sequence or encoding the indicated substitutions of residue G70. Cells were grown at indicated temperatures for 2 days. Dashed line indicates that two plates were used; cells were grown, plated and incubated side by side. (*right*) Comparison of Ydj1 levels in strains expressing *ydj1*_*134*_ variants; in addition, WT *YDJ*_*134*_ expressed in *ydj1-Δ* strain is shown as an expression control at far left. Cell extracts were subjected to immunoblot analysis using antibodies against the J-domain of Ydj1 or, as a control, Ssc1.

Hsp70s are two-domain, allosteric machines. The N-terminal nucleotide binding domain (NBD) contains the ATPase catalytic site; the C-terminal substrate binding domain (SBD) contains the peptide binding pocket; the two domains are connected by a flexible linker (**Fig 1A**)[3, 6–8]. The SBD consists of two subdomains: one, called SBDβ, contains the peptide-binding pocket; the other, SBDα, can position as a 'lid' over the pocket trapping the substrate. The ATP- and ADP-bound Hsp70 conformations are very different. When ATP is bound, both SBDα and SBDβ, as well as the flexible linker, interact with the NBD [9, 10]. In this way, the lid is held away from the peptide binding pocket, allowing substrate free access. The J-domain of J-proteins, which is responsible for ATPase stimulation, interacts at the interface of the NBD and SBDβ. The critical, invariant HPD tripeptide is in the loop between the two longest (II and III) of the J-domain’s four helices. Upon ATP hydrolysis, the SBD and the linker dissociate from the NBD. Unrestrained, the α-helical lid covers the peptide binding pocket, hindering substrate access or, if substrate was interacting at the time of ATP hydrolysis, hindering its dissociation [11, 12]. Relevant to this report, these states are likely not as all-or-none as outlined above. Recent biophysical results have indicated that the cycle is much more dynamic than this description implies [13, 14]. The Hsp70 conformations described above are the predominant, but not the sole, states. In addition, transient intermediates exist, mostly due to the mobility of the lid, which upon interaction of substrate in the peptide-binding cleft, partially dissociates from the NBD (**Fig 1A**).

Apart from the J-domain, J-proteins are very structurally heterogeneous. The most ubiquitous and abundant are those having a double β-barrel substrate binding domain of which there are two general types [15]. One, which includes *Escherichia coli* DnaJ and *Saccharomyces cerevisiae* Ydj1, as well as its human homolog DNAJA1, have a zinc-binding domain (ZnBD) within the double β-barrel segment. These are often called Class I or Class A J-proteins [16, 17]. The second, which includes Sis1 of *S*. *cerevisiae* and its human homolog DNAJB1, does not have a ZnBD. These are often called Class II or Class B J-proteins [16, 17]. All double β-barrel proteins have a glycine (Gly)-rich region between the N-terminal J-domain and the β-barrels, which is often called the G/F region as it is typically rich in both phenylalanine and glycine [2].

Although significant progress has been made regarding J-protein co-chaperone function, major unanswered questions remain, particularly regarding the ability of J-proteins to drive specific functions of Hsp70-based chaperone machinery. Sis1 is a prime example. It is essential even under optimal growth conditions [18] and is required for the maintenance of yeast prions [19, 20]. None of the other 12 J-proteins of the cytosol or nucleus can substitute for Sis1 to rescue viability or support prion maintenance, even the more abundant and structurally similar Ydj1 which partners with the same Hsp70 [19, 21]. However, Sis1 homologs from other species, including human DnaJB1, can substitute, indicating functional conservation in evolution [22]. Moreover, the N-terminal segment of both Sis1 and DnaJB1, which contains the J-domain and Gly-rich region, is sufficient to sustain growth and maintain at least some prions [23–25]. The mechanism behind this ability is not understood, as this segment lacks the β-barrel substrate binding domain.

Why Sis1 is uniquely essential is not known. Neither the essential cellular processes in which its involvement is required, nor the substrates with which it interacts are identified. To approach these issues we took a genetic approach, isolating mutations that overcome the Sis1 requirement for cell growth. Surprisingly, rather than uncovering a Sis1 essential substrate or specific cellular process required for Sis1 function, we isolated mutations in *YDJ1* encoding amino acid substitutions near the end of the J-domain. We reasoned that the position and biochemical effect of these gain-of-function substitutions likely affected functional interaction with Hsp70 rather than either substrate binding per se or sub-localization within the cytosol or nucleus. To test this hypothesis, we set up a selection for suppressor mutations in *SSA1*, the gene encoding the partner Hsp70 of both Ydj1 and Sis1. Substitution mutations encoding alterations in the SBDβ of Ssa1 that affect its interaction with the SBDα lid were isolated. Together, our data point to the idea that fine tuning of the balance between conformation states of Hsp70 plays a pivotal role in Hsp70’s ability to productively interact with certain substrates. We suggest that such tuning of the initiation of the substrate interaction cycle promotes diversity of Hsp70 system function.

## Results

### Substitutions in Ydj1 suppress the lethality caused by absence of Sis1

To better understand the specificity of Sis1 function, we selected for spontaneous genomic mutations that permit growth of cells having a deletion of *SIS1* (*sis1-Δ*). *sis1-Δ* cells expressing Sis1 from a *URA3*-based plasmid were plated on media containing 5-fluoroorotic acid (5-FOA), which is toxic to cells expressing the *URA3* gene. The strongest suppressor strain obtained formed colonies at 23, 30 and 34°C, although growth was considerably less robust than that of cells expressing Sis1 (**Fig 1B and 1D**).

During backcrossing, we observed that the suppressing mutation showed strong linkage with the *SIS1* locus. Noting that *YDJ1* and *SIS1* are only 111 kb apart on the chromosome, we used a candidate approach, reasoning that, as these two J-proteins have structural similarities (**Fig 1C**), an alteration in Ydj1 might allow it to substitute for Sis1. We cloned the *YDJ1* open reading frame from the suppressor strain (*YDJ1*_*SUP*_), placing it under the control of either the heterologous TEF or native *YDJ1* promoter in centromeric plasmids. *sis1-Δ* cells expressing the *YDJ1* gene isolated from the suppressor strain formed colonies at 23 and 30°C (**Fig 1D**). Next, we constructed a strain in which both the *YDJ1* and *SIS1* genes on the chromosome were deleted, so that the only Ydj1 present in the strain was that expressed from the gene cloned from the suppressor strain. Growth of these cells, which expresses Ydj1_SUP_ at a level similar to that of Ydj1 in wild-type (WT) cells, was as robust as that of the originally isolated suppressor strain, suggesting that the *YDJ1*_*SUP*_ allele, like many gain of function mutations, is semidominant, perhaps due to competition with WT protein. In sum, we conclude that growth of the initial viable *sis1-Δ* isolate is due to a mutation(s) in the *YDJ1* gene.

Sequencing of the *YDJ1* gene isolated from the suppressor strain revealed a single G to A base substitution, resulting in change of the glycine codon at position 70 to a serine codon. G70 is at the junction between the J-domain and Gly-rich region. Because the N-terminal segment of Sis1 lacking the substrate-binding β-barrel CTDs is sufficient for cell growth [23], we tested whether this was also the case for the suppressive ability of G70S substitution in Ydj1. An N-terminal 134 residue fragment of Ydj1 containing the G70S substitution (Ydj1_134_G70S) supported growth of *sis1-Δ* cells (**Fig 1E**). Noting that serine is both more bulky and polar than glycine, which lacks a side chain, we tested the effect on suppression of substitutions at position 70 by 5 other amino acids (A, V, T, L and N) having varying degrees of bulkiness and polarity. All variants were expressed at very similar levels. All allowed growth of the *sis1-*Δ strain to some extent. Among all the variants, G70N supported the most robust cell growth and was used for further experiments.

### The G70N substitution alters conformation at the J-domain and Gly-rich region junction

**Fig 2:**
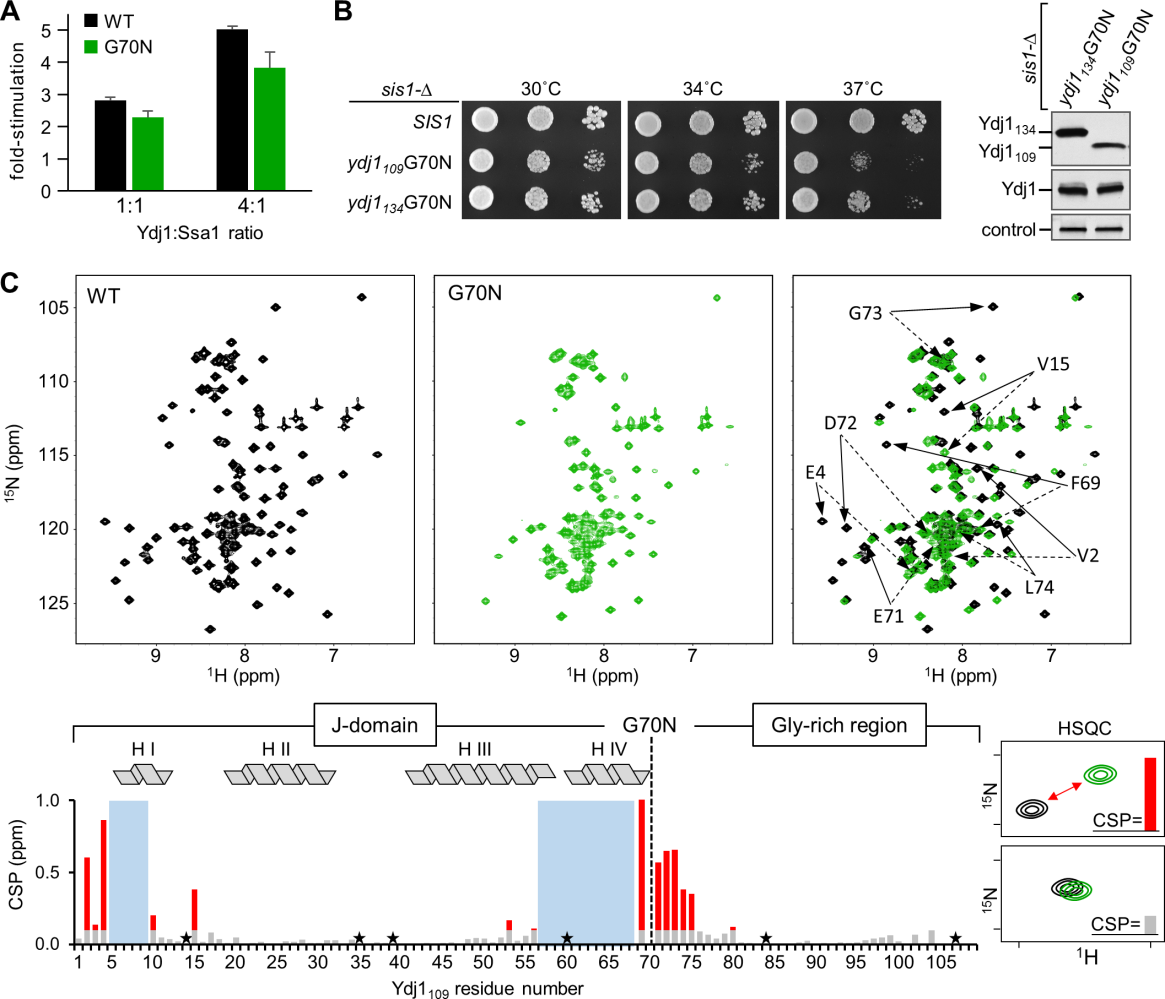
G70N substitution in Ydj1 affects structural properties around the junction of J-domain and Gly-rich region. (**A**) Single turnover ATPase assays were performed using ^32^P-ATP/Ssa1 complex. Data are presented as fold-stimulation at indicated ratios of Ydj1 or Ydj1G70N, relative to basal Ssa1activity. Error bars indicate the standard deviation. The mean of 6 experiments is plotted. (**B**) (*left*) 10-fold serial dilutions of cells lacking Sis1 (*sis1-*Δ) carrying a plasmid with an insert encoding WT Sis1 (*SIS1*) or the 109 or 134 residue N-terminal Ydj1 fragment with the G70N substitution (*ydj1*_*109*_*G70N* and *ydj1*_*134*_*G70N*, respectively). (*right*) Lysates made from these strains were subjected to immunoblot analysis using antibodies specific for the Ydj1 J-domain and, as a control, Ssc1. (**C**) NMR analysis of conformational changes in Ydj1_109_ due to G70N substitution. (*top*) Comparison of 2D ^1^H,^15^N HSQC spectra obtained for Ydj1_109_WT (black) and Ydj1_109_G70N (green). The signal position of each residue is defined by chemical shifts along the ^15^N (y-axis) and ^1^H (x-axis) dimension, which reflects protein conformation. (*right*) Overlay of the two HSQC spectra. Most signals are very similar, with a number of signals exhibit substantial differences in position. Arrows indicate signals of the 8 most affected residues: WT (solid), G70N (dotted). (*bottom)* Histogram representing differences in each residue’s signal position in Ydj1_109_WT and Ydj1_109_G70N HSQC spectra. Change indicated by height of bar, calculated as the combined difference in chemical shifts between signals for each residue. The magnitudes of the chemical shift perturbations (CSP) are color-coded: (red) CSP >0.1 ppm; (gray) CSP ≤ 0.1 ppm; (blue rectangles) signals from residues T5-D9 and L57-Q68 that were identified in the Ydj1_109_WT spectrum, but not in Ydj1_109_G70N; (star) proline residues, which have no signal in HSQC spectra. The α-helices of the J-domain are indicated at the top of the panel. The G70N substitution is indicated by a dotted line.

To investigate how a change at the end of the J-domain affects function and structure, we compared WT Ydj1 and Ydj1 having the G70N substitution using two approaches: ATPase assays and nuclear magnetic resonance (NMR) spectroscopy. To assess stimulation of Ssa1 ATPase activity we used preformed ^32^P-ATP-Ssa1 complexes to carry out single-turnover assays. Both Ydj1 proteins stimulated Ssa1’s ATPase activity. Ydj1 with the G70N substitution was modestly less effective, being on the order of 20% less active than WT protein (**Fig 2A**).

NMR is most effectively performed on small proteins. Therefore, before beginning structural analysis, we tested whether the N-terminal, 109 residue fragment, which encompasses the entire J-domain and Gly-rich region (Ydj1_109_G70N), was able to support growth of *sis1-Δ* cells (**Fig 2B**). Even though present at a level several fold lower than the 134 residue fragment used in the experiments described in the previous section, Ydj1_109_G70N was able to support colony formation at 30 and 34°C. We then collected ^1^H-^15^N HSQC spectra for the 109 residue WT and the G70N fragments. We chose this approach, as the position of the signal for each residue in the spectra, which arises from the backbone amide, depends not only on the type of residue, but also its surrounding chemical environment. As a result, comparison of signal position arising from the same residue in the WT and the G70N variant provides insights into conformational similarities and differences. We successfully identified signals for nearly all Ydj1_109_WT residues, including those for the 16 glycines of the Gly-rich region, many of which are overlapping (**Fig 2C**). Comparison of the two spectra revealed that of the 101 assigned WT signals, 71 from G70N had comparable positions, suggesting similar conformations of these regions. In the J-domain, these signals correspond to residues concentrated in helix II, the loop containing the HPD motif, and the adjacent N-terminal segment of helix III. Other signals with comparable positions came from residues of the C-terminal segment of the Gly-rich region.

Of the 30 other WT residues for which we assigned signals, 14 counterparts were assigned in the G70N spectra. All of these exhibited large (>0.1 ppm) changes in position. Signals from the remaining 16 residues were not detected. Their lack of resolution is likely because they undergo movement on a time scale that hinders detection, and thus also indicative of differences in the local conformation between G70N and WT. These 30 residues clustered in two regions of the linear Ydj1_109_ sequence (**Fig 2C**): one, in the interval including the end of the J-domain and beginning of the Gly-rich region, encompasses the end of helix III, through helix IV and the first residues of the glycine-rich region; the other contains N-terminal residues and helix I. Changes in the signals of immediate neighbors of residue 70 were expected, because of the chemical differences between glycine and asparagine. However, both the number of affected residues and the magnitude of the changes observed in the HSQC signal pattern suggest a local conformational change in the structure of the J-domain and its junction with Gly-rich region. Nonetheless, no major changes were found in the regions known to be critical for stimulation of Hsp70’s ATPase activity.

### Substitutions of Y66 of Ydj1 allow growth of *sis1-Δ*

**Fig 3:**
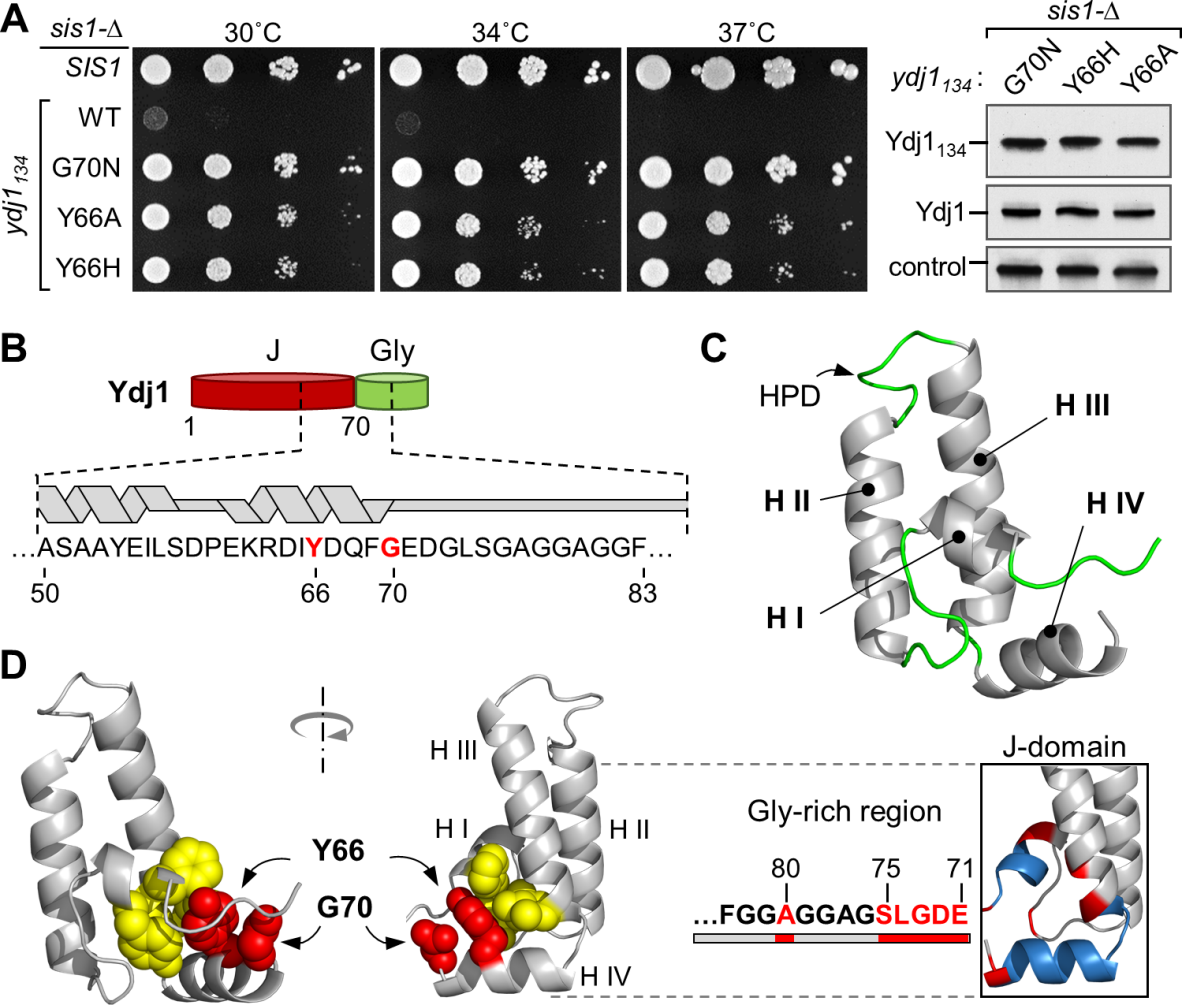
Substitutions of Y66 in helix IV of the J-domain allow Ydj1_134_ to support growth of *sis1-Δ*. (**A**) (*left*) Cells lacking *SIS1* (*sis1-*Δ), harboring a plasmid carrying an insert encoding WT Sis1 (*SIS1*), or Ydj1_134_ with no substitutions (WT) or indicated substitution, were plated as 1:10 serial dilutions and grown at 30 or 34°C for 2 days, or at 37°C for 3 days. (*right*) Lysates prepared from the strains were subjected to immunoblotting using antibodies against the J-domain of Ydj1 or Ssc1 (control). (**B**) Diagram of Ydj1 J-domain/Gly-rich region with sequence and position of helices in segment encompassing residues 50–85 indicated; suppressor positions Y66 and G70 in red. (**C**) Averaged NMR structure of Ydj1 J-domain (N-terminal 70 residues) generated from coordinates of 20 lowest-energy conformers presented as ribbon representation and colored by secondary structure (gray for α-helix, green for unstructured regions). (**D**) (*left*) Ydj1 J-domain structure with Y66 and G70 (red) and residues with which Y66 interacts (yellow; F7, Y8, I56, L57) in sphere representation. (*right*) Most affected residues (i.e. those with the largest CSPs (red) and those missing signals in Ydj1_109_G70N (blue) as shown in Fig 3C) mapped on Ydj1 J-domain structure and in the sequence of the Gly-rich region.

A single Ydj1 isolate (i.e. a substitution of G70) was isolated in our initial search for spontaneous suppressors of the inviability of *sis1-Δ*. We therefore carried out a genetic selection targeted to *YDJ1* to identify substitutions at other positions that allow growth of *sis1-Δ*. Using a library of *YDJ1*_*134*_-containing plasmids constructed using error-prone PCR, we selected for transformants able to grow in the presence of 5-FOA. We did recover *ydj1*_*134*_ alleles at position 70, specifically S and D substitution mutations. The other isolate that approached the ability of Ydj1_134_G70N to support growth of cells lacking Sis1 encoded H in place of Y at position 66 (**Fig 3A**). Suppression by substitution at Y66 is not unique to histidine; Ydj1_134_Y66A suppressed similarly to Ydj1_134_Y66H.

Y66 is in Helix IV (**Fig 3B**), the helix having the most poorly resolved position in other NMR structures of Class I/A J-domains, due to low number of contacts made with the rest of the protein. To better place Y66 in Ydj1’s J-domain, we determined the structure of the N-terminal 70 residue fragment (Ydj1_70_) using NMR (**Fig 3C**; **S1 Table**). The J-domain of Ydj1 has an overall fold that strongly resembles that of other J-domains (e.g. PDB: 1XBL, 2M6Y, 1HDJ). The two longest helices interact with each other, connected by the solvent exposed loop containing the invariant HPD motif, with the two shorter helices, I and IV, oriented towards the same face of helix II/III. A number of hydrophobic contacts between helices I, II and III stabilize the general fold of the J-domain and contacts between helix IV and the rest of the protein are much more limited. We found that Y66 anchors helix IV to the hydrophobic core via interactions with residues of both helix I and helix III, emphasizing the importance of this residue for determination of helix IV orientation (**Fig 3D**).

In addition, G70 directly faces Y66 in the solved structure (**Fig 3D**). The structure suggests that substitutions at position 70 might directly affect Y66 and thus the orientation of helix IV. Indeed, the pattern of changes observed by NMR for the G70N variant compared to WT is consistent with an alteration of the contact network between Y66 and the rest of the protein. Even residues with observed changes like A53 of helix III or V15 in the loop between helix I and II, which are isolated from the bulk of affected regions (**Fig 2C**), can be explained by their close proximity to helix IV (**Fig 3D**). Taken together results of both genetic and structural analysis suggest that single residue substitutions in helix IV are sufficient to “unlock” Ydj1’s ability to compensate for Sis1 function. Such substitutions affect local conformation at the junction of the J-domain and Gly-rich region, without strongly affecting its site of interaction with Hsp70.

### G70N and Y66H Sis1 variants support maintenance of a strong [*PSI*^*+*^] prion variant

**Fig 4:**
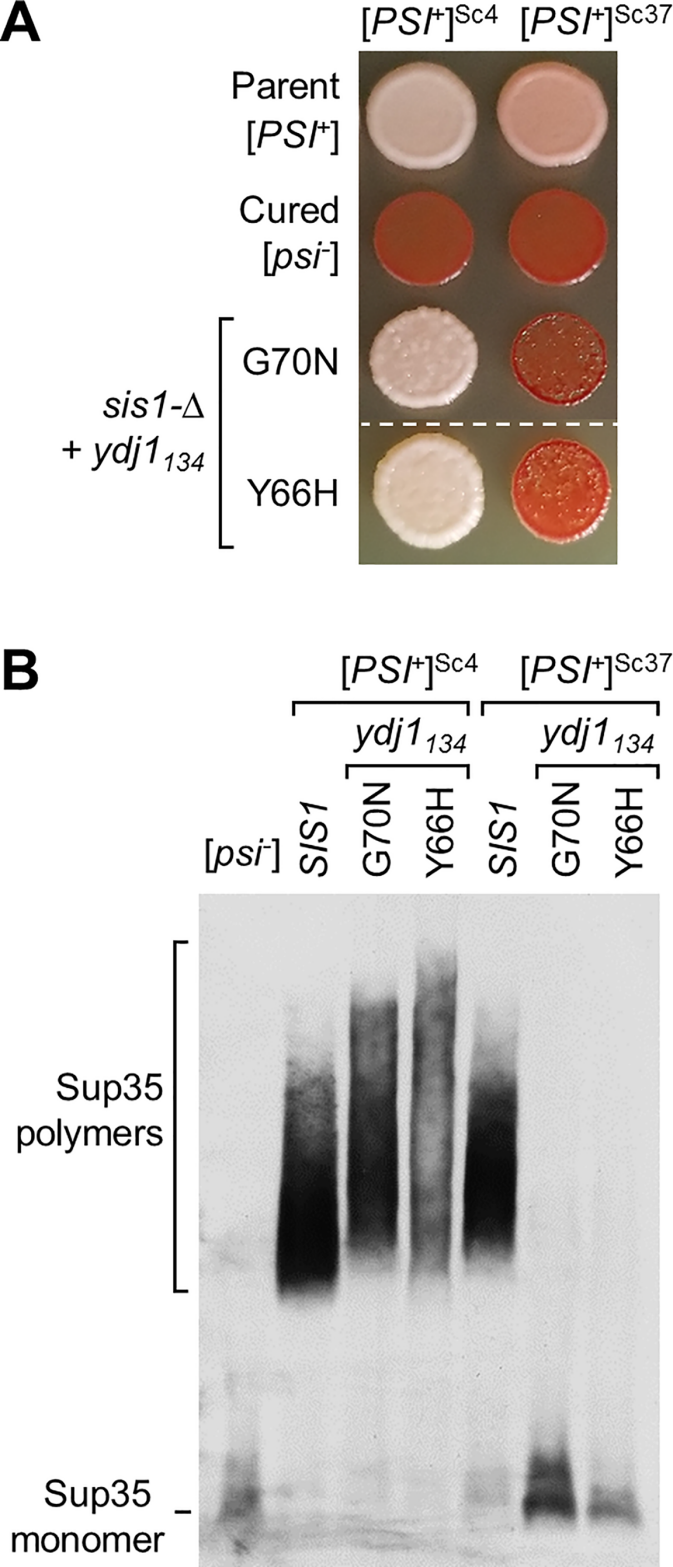
G70N and Y66H substitutions in Ydj1 allow maintenance of the strong [*PSI*^*+*^] prion. (**A**) Maintenance of Sc4 (strong) and Sc37 (weak) [*PSI*^*+*^] by Ydj1_134_ suppressor variants in *sis1-Δ* strains. Equal number of cells were dropped on YPD and grown at 23˚C for 5 days for assessment of color development. Dashed line indicates different parts of the same plate where irrelevant strains were cropped out of the image. For both variants, a representative of the 10 candidates isolated from 5-FOA plates that were tested for [*PSI*^*+*^] maintenance; all showed similar color phenotype. Parent [*PSI*^*+*^] cells for each prion variant (parent) and cells cured of the prion by growth in the presence of GdnHCL (cured). (**B**) For analysis of [*PSI*^*+*^], cell lysates were prepared, resolved by SDD-AGE and subjected to immunoblotting using Sup35-specific antibodies.

As Sis1 is required for maintenance of yeast prions, we tested the ability of the *YDJ1* suppressors to maintain two prions, [*PSI*^*+*^] and [*RNQ*^+^], formed by the translation termination factor Sup35 and Rnq1 proteins, respectively. A single prion-forming protein, such as Sup35, can form multiple prion variants, depending on the particular amyloid conformation formed. Such variants are often referred to as “strong” or “weak”, depending on phenotypic intensity and their stability through cell divisions. We tested two [*PSI*^*+*^] variants—one strong ([*PSI*^*+*^]^Sc4^) and one weak ([*PSI*^*+*^]^Sc37^). *sis1-Δ* cells having either variant of the [*PSI*^+^] prion and expressing WT Sis1 from a *URA3* plasmid were transformed with a plasmid carrying Ydj1_134_G70N or Ydj1_134_Y66H. Cells having lost the *URA3* plasmid were then selected on 5-FOA plates. These strains have a nonsense allele of *ADE1*, a gene required for adenine synthesis. When Sup35 is in the soluble state, efficient translation termination results in an accumulation of a red pigmented intermediate [26]. However, when the Sup35 is in the prion form, translation termination is inefficient, causing the cells to appear white or light pink due to the reduction in pigment accumulation. Patches of cells having the strong [*PSI*^*+*^]^Sc4^ variants were white after extended growth, while those having weak [*PSI*^*+*^]^Sc37^ were red (**Fig 4A**), suggesting maintenance of the strong variant, but not the weak variant. To verify this interpretation, we carried out a biochemical assay, semi-denaturing detergent agarose gel electrophoresis (SDD-AGE), in which large detergent-resistant polymers can be resolved and visualized by immunoblot analysis [27]. As expected large Sup35 polymers were resolved in the strong, but not the weak, [*PSI*^*+*^] Ydj1_134_G70N or Ydj1_134_Y66H strains (**Fig 4B**). The polymer size in cells expressing the Ydj1 variants was larger than those in cells expressing Sis1, indicative of less efficient fragmentation of prion fibrils. We also tested the maintenance of [*RNQ*^+^]. Neither Ydj1 variant maintained this prion, as indicated by the lack of detergent-resistant Rnq1 aggregates (**S1A Fig**).

### Hsp70 *SSA1* suppressors of *sis1-Δ* inviability

**Fig 5:**
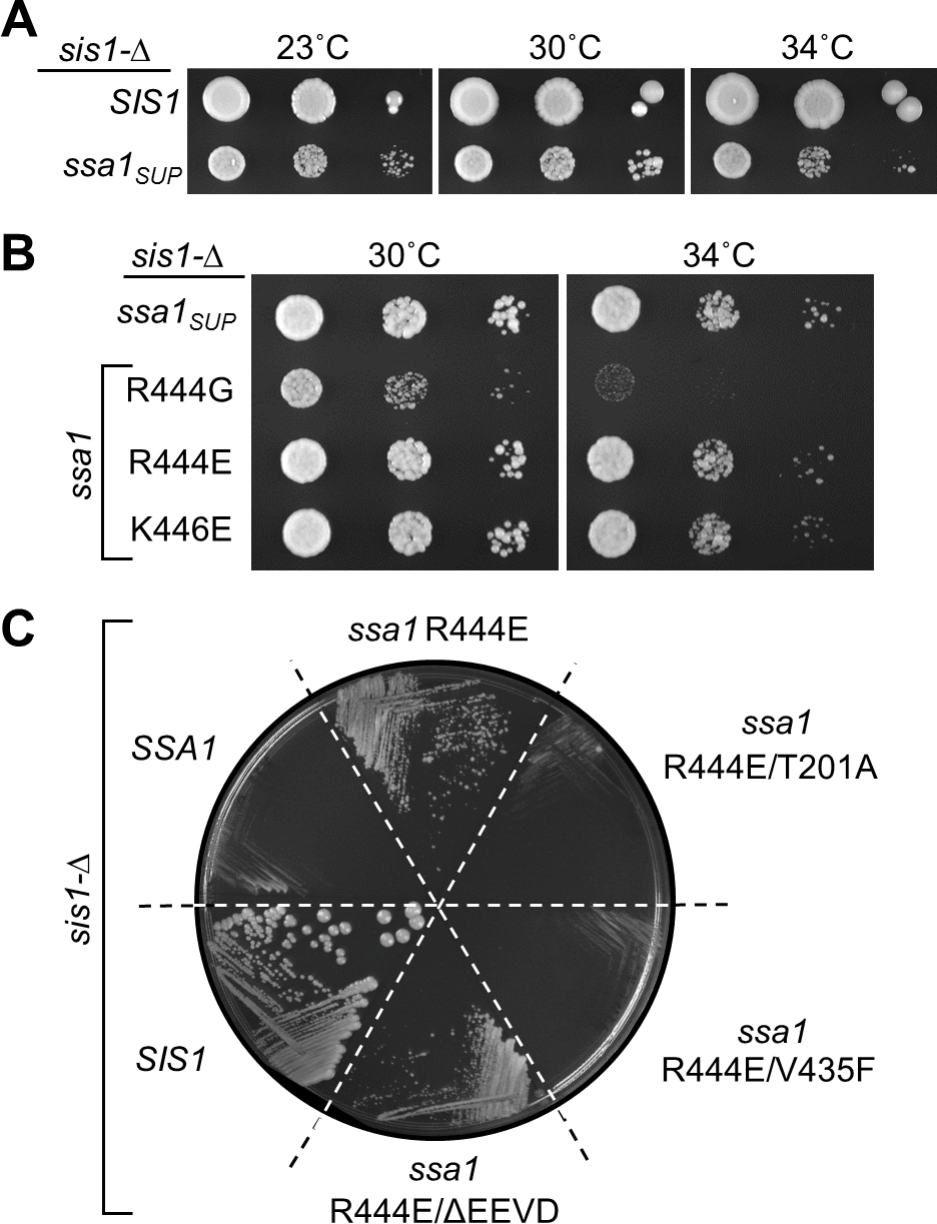
Substitutions in Ssa1 allow growth of *sis1-Δ* cells. (**A,B**) Ten-fold serial dilutions of *sis1-Δ* strains harboring a plasmid with an insert of a WT *SIS1* gene (*SIS1*), the originally isolated *SSA1* suppressor mutant (*ssa1*_*SUP*_) or the indicated *SSA1* substitutions. Plates were incubated at indicated temperatures for 4 days. (**C**) *sis1-Δ* cells harboring a plasmid with a WT *SIS1* gene and *URA3* marker plus the indicated additional *TRP1*-marked plasmid were streaked onto plates containing 5-FOA and incubated for 5 days at 30°C. *TRP1* plasmids had inserts encoding WT (*SIS1*), or *SSA1* genes, either WT (*SSA1*) or indicated variants. ΔEEVD indicates that the C-terminal four residues are absent.

The position of the Y66 and G70 residues suggested to us that neither established mode of suppression, that is, neither direct interaction with substrate nor subcellular localization, was responsible for suppression by the isolated Ydj1 variants. Rather, we hypothesized that suppression involved J-domain functioning, and thereby affecting Hsp70’s interaction with substrate. We reasoned that if this is the case, alterations in Hsp70 itself might also allow cells to grow in the absence of Sis1. To test this idea, we undertook a genetic selection for mutations in the HSP70 gene, *SSA1*, which allowed colony formation by *sis1-Δ*, by transforming with a randomly mutagenized *SSA1* plasmid library, followed by plating on 5-FOA to select for loss of the *URA3*-based WT *SIS1* plasmid. One *SSA1* suppressor gene (*ssa1*_*SUP*_) was isolated (**Fig 5A**). *ssa1*_*SUP*_ contains mutations encoding seven amino acid substitutions distributed throughout the protein. Seven *ssa1* genes, each containing one of the mutations, were constructed. Only one of the substitutions, R444G, allowed growth of *sis1-Δ* (**Fig 5B**). A reversal of charge at this position had a more dramatic effect than the G substitution present in the initial isolate, as Ssa1_R444E_ was more effective at suppression than Ssa1_R444G_; *sis1-Δ* cells expressing Ssa1_R444E_ formed single colonies even at 34°C. Like *ydj1*_*134*_*G70N*, cells expressing *ssa1*_*R444E*_ were not able to support maintenance of [*RNQ*^*+*^] or [*PSI*^*+*^]^Sc37^ (**S1B Fig**). Unfortunately, we were not able to confidently test maintenance of strong [*PSI*^*+*^]^Sc4^ because of its severe effect on the rate of growth of these cells.

We next asked if fundamental activities important for Hsp70 function were required for suppressive activity. We combined R444E with one of two loss-of-function substitutions, in either the catalytic ATPase site (T201A) or in the peptide-binding site (V435F) [28, 29]. Neither Ssa1_T201A/R444E_ nor Ssa1_V435F/R444E_ allowed growth of *sis1-Δ* cells (**Fig 5C**). We also tested the importance of the conserved tetrapeptide EEVD, which is at the extreme C-terminus of all eukaryotic cytosolic Hsp70s such as Ssa1, and interacts with cellular proteins with which cytosolic Hsp70s functionally cooperate [30, 31]. *sis1-Δ* cells expressing Ssa1_R444E/ΔEEVD_ grew nearly as well as those expressing Ssa1_R444E_ (**Fig 5C**). Together, the data suggest that suppression by the R444E substitution depends on the universal features of Hsp70s (ATPase activity and peptide binding cleft), but not the C-terminal EEVD.

### Ssa1 suppressor substitutions affect interaction between SBDβ and SBDα lid

**Fig 6:**
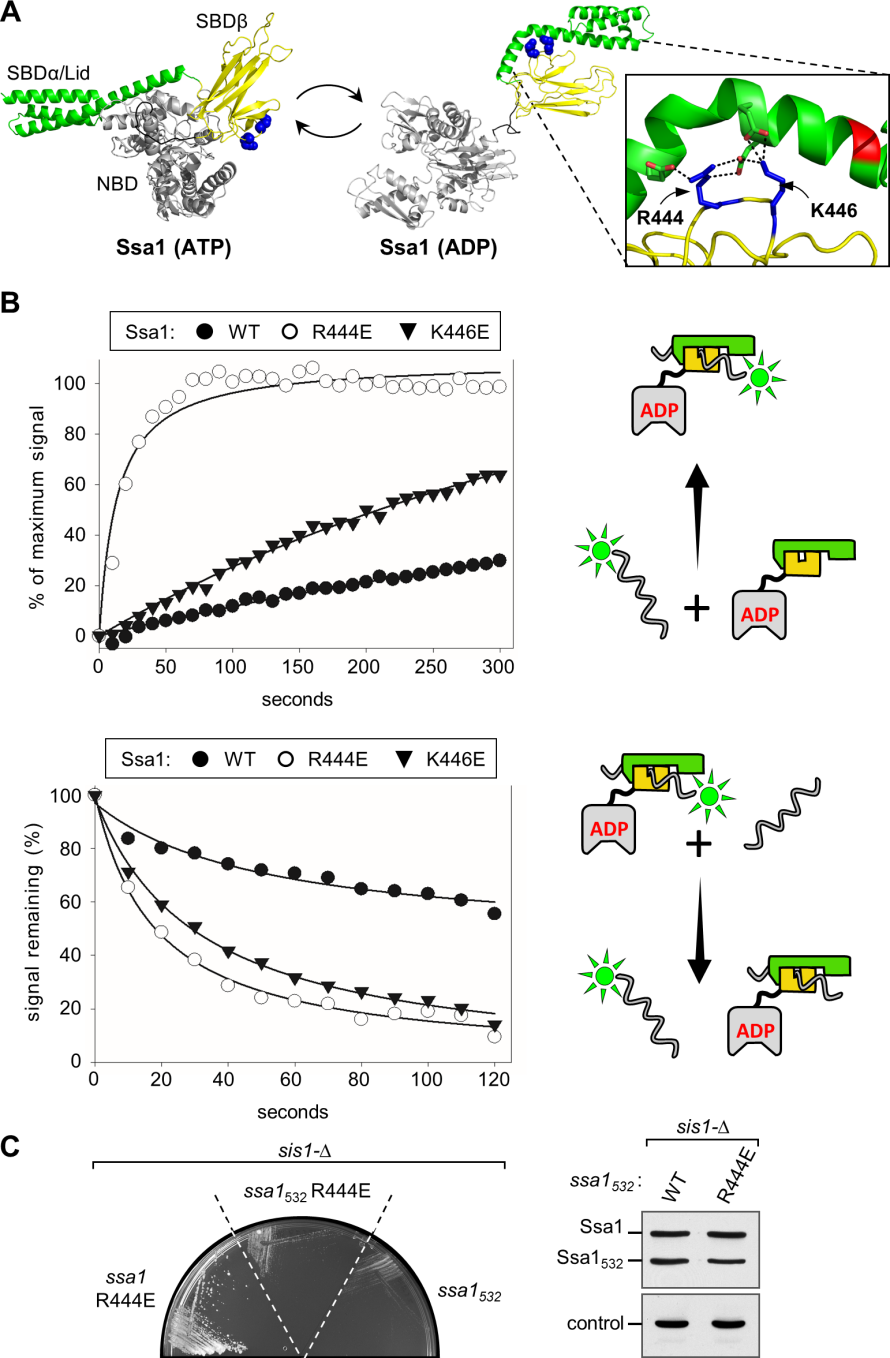
Interaction of Ssa1 variants with peptide substrate. (**A**) Models of Ssa1 structures in the ATP- and ADP-bound states. Nucleotide binding domain (NBD), gray; α- and β-subdomains of substrate binding domain (SBD), green and yellow, respectively. Sidechains of R444 and K446 shown in sphere representation (blue). Homology modeling was performed using SWISS-MODEL; DnaK structures, PDB: 2KHO and 4JN4, used as templates for ADP and ATP states, respectively. In the zoomed-in view, the sidechains of R444, K446 (blue) and the residues with which they interact (E518/E525 and E525/D526, respectively) are shown in stick representation, with oxygen (red); interaction indicated by dotted lines. (**B**) Interaction of peptide substrate with WT and variant Hsp70s using fluorescin-labeled P5 peptide, measured by fluorescence anisotropy. Cartoons at right depict reactions: Hsp70 domains: NBD, gray; SBDβ, yellow; SBDα, green. Peptide, wavy line; fluorescein label, neon green. (*Top*) Kinetics of Ssa1/F-P5 complex formation. Left, at t = 0, 10 nM fluorescein labeled P5 (F-P5) and 5 μM Ssa1 WT or variants were combined in the presence of 1 mM ADP. Fluorescence polarization data were collected over time and plotted as a percent of binding observed at equilibrium in assays in which incubation was extended overnight. Each data point represents the mean of three experiments. (*Bottom*) Kinetics of peptide dissociation. F-P5 and Ssa1 were combined to yield reaction compositions identical to those described in (B) and incubated until equilibrium was reached. At t = 0, excess P5 was added and fluorescence polarization monitored over time. Values are plotted as a percent of the initial signal remaining. Each data point represents the mean of three experiments. Right, schematic of assay. (**C**) (*left*) Growth of *sis1-Δ* cells harboring a plasmid with a WT *SIS1* gene and *URA3* marker plus the indicated *TRP1*-marked plasmids encoding full-length Ssa1 or Ssa1 lid-truncated variants containing N-terminal 1–532 residues (Ssa1_1-532_) with or without an additional R444E substitution. Site of the SBDα truncation indicated in red in (A). (*right*) Cell lysates of starting strains expressing WT Ssa1 and truncated *ssa1*_*1-532*_ variants were subjected to immunoblot analysis using antibodies raised against Ssa1 or, as a control, Tim44.

R444 is a highly conserved residue in SBDβ. Based on modeled Ssa1 structures obtained using DnaK of *E*. *coli* as a template, R444 interacts with negatively charged residues of the SBDα lid, participating in the stabilization of its closure over the peptide binding pocket in the ADP state (**Fig 6A)**. But, it is not engaged in an intramolecular interaction in the ATP state. The enhanced suppression by Ssa1_R444E_ compared to Ssa1_R444G_ is consistent with this. To further test this idea, we altered another SBDβ residue, K446, predicted to interact with the lid. Consistent with this idea, Ssa1_K446E_ was more effective at suppression of the *sis1-Δ* growth defect than Ssa1_R444G_, supporting colony formation at 34°C and below, similar to Ssa1_R444E_ (**Fig 5B**).

If the substitutions decrease interaction between SSBα and SSBβ, both the on- and off-rates of substrate when Hsp70 is bound to ADP would be expected to increase. This is because interaction of the SBDα lid with the SBDβ subdomain impedes access of substrate to the cleft and also impedes substrate dissociation once the lid is closed. To evaluate substrate binding we used fluorescence anisotropy, comparing interaction of WT Ssa1, Ssa1_R444E_ and Ssa1_K446E_ with fluorescein-tagged peptide substrate P5 (F-P5) in the presence of ADP. Association kinetics were assessed by measuring the increase in anisotropy with time after addition of F-P5 (**Fig 6B; S2 Fig**). As expected, as a consequence of the lid inhibiting access of substrate to the cleft in SBDβ, binding of F-P5 to WT Ssa1 occurred slowly, reaching only ~20% of maximal binding at 5 min. In contrast, interaction of the variants with substrate was more rapid. In the case of Ssa1_R444E_, 50% of maximal binding occurred within 11 seconds. Binding to Ssa1_K446E_ was also faster than to WT Ssa1; by 5 min ~60% maximal binding was attained. To compare dissociation rates, Ssa1/F-P5 complexes were preformed. Excess unlabeled P5 was then added and the decrease in anisotropy monitored (**Fig 6B**). In the case of WT protein, the signal had decreased by approximately 50% at the 420 second point. F-P5 dissociation from the two variants was more rapid. After addition of unlabeled peptide, the anisotropy signal decreased 50% within 18 and 29 seconds in the case of Ssa1_R444E_ and Ssa1_K446E_, respectively. Together these data are consistent with the substitutions destabilizing the interaction of the lid and SBDβ, resulting in both faster on- and faster off-rates of peptide substrate in the presence of ADP.

As these results indicate that the stability of the lid-SBDβ interaction is reduced, we tested if the lid is important for suppression. The C-terminal truncation, Ssa1_1-532_, lacking 110 residues, including those that cover the peptide binding cleft, did not allow growth of *sis1-Δ*, either alone or in combination with R444E, even though the protein is expressed at levels comparable to WT Ssa proteins (**Fig 6C**). Thus, the results suggest that modulation, not elimination, of the lid-SBDβ interaction is important for suppression.

### Ssa1 suppressor substitutions affect ATPase stimulation by peptide substrate

**Fig 7:**
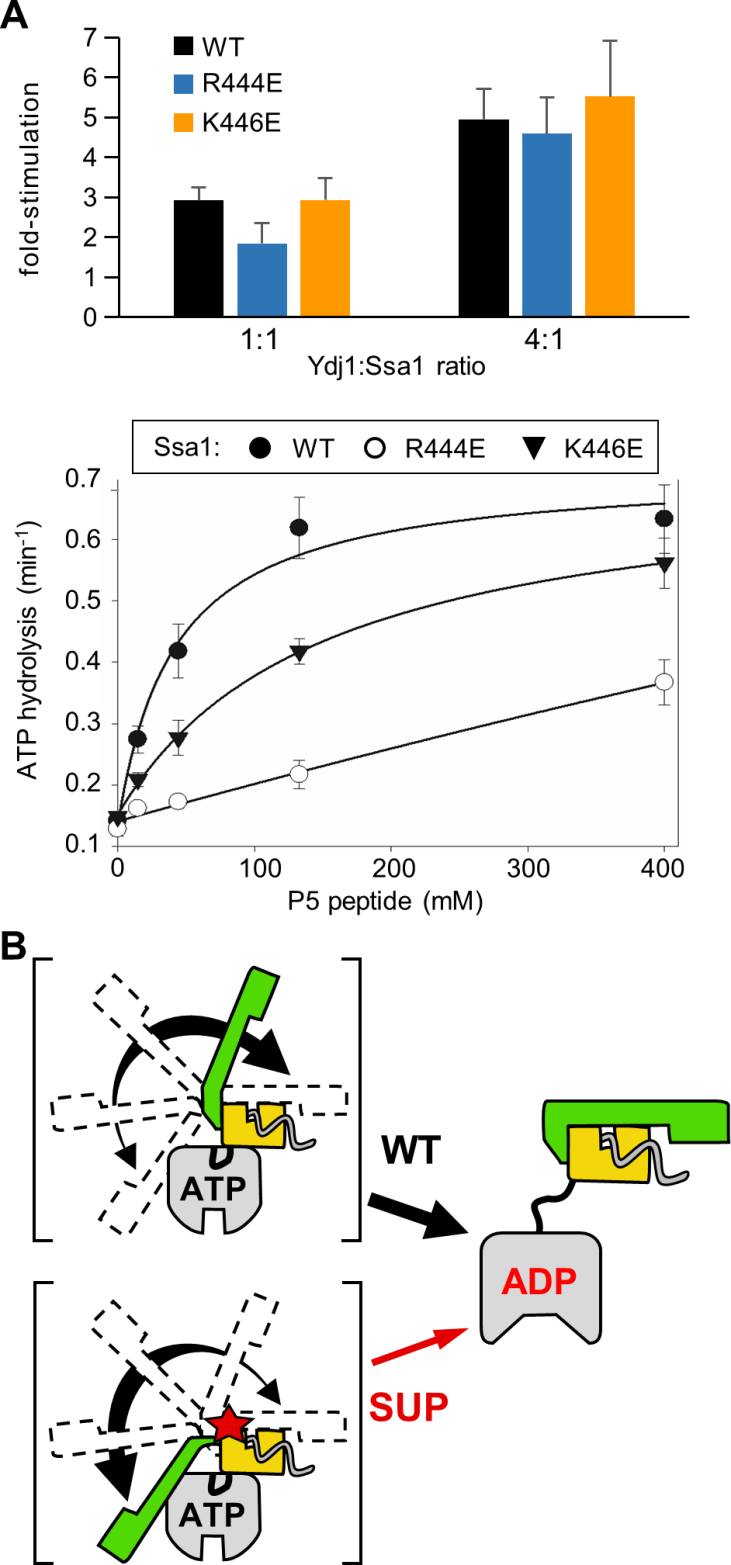
Stimulation of Ssa1 ATPase activity by peptide substrate, but not Ydj1, is reduced in R444E and K446E variants. (**A**) Single turnover ATPase assays were performed using WT and variants Ssa1 ^32^P-ATP complexes. Data are presented as fold-stimulation relative to the basal activity for each Ssa1 protein. Error bars indicate the standard deviation. (*top*) stimulation by Ydj1. The mean of 6 experiments is plotted. (*bottom*) Stimulation by peptide P5. Assays were performed at four P5 concentrations and apparent rate constants (min^-1^) calculated. The mean of three experiments is plotted. Basal turnover rate (min^-1^): Ssa1, 0.141 +/- 0.003; Ssa1_R444E_, 0.128; Ssa1_K446E_, 0.148 +/- 0.007. (**B**) Schematic representation of the transient intermediates of Hsp70-substrate interaction. Dotted lines in II indicate detachment of SBDα from NBD. Dotted lines in I and III indicate: SBDα transiently detaches from NBD in ATP-state in the absence of substrate interaction (I) and transiently from the SBDβ in the ADP-state (III).

We compared the ability of both Ydj1 and peptide substrate to stimulate the ATPase activity of WT and variant Ssa1. The basal ATPase activities of the three proteins were similar, differing by less than 2 fold. Also, little difference in stimulation between WT and variants was observed upon Ydj1 addition (**Fig 7A**). However, peptide substrate stimulated the two variants less effectively than WT Ssa1 over a range of concentrations, with Ssa1_R444E_ being the more defective of the two variants. For example, Ssa1_R444E_ was stimulated 1.7 fold at a peptide concentration of 133 μM, compared to 2.8 fold and 4.4 fold in the case of Ssa1_K446E_ and WT, respectively. Together these results indicate that the interaction of the SBDα lid with SBDβ is destabilized by substitutions at position 444 or 446 (**Fig 7B**). As discussed below, this altered interaction may also affect the kinetics of trapping of substrate as indicated by the reduced stimulation of the variants by peptide substrate.

### General in vivo functionality of Ssa1 and Ydj1 suppressor variants

**Fig 8:**
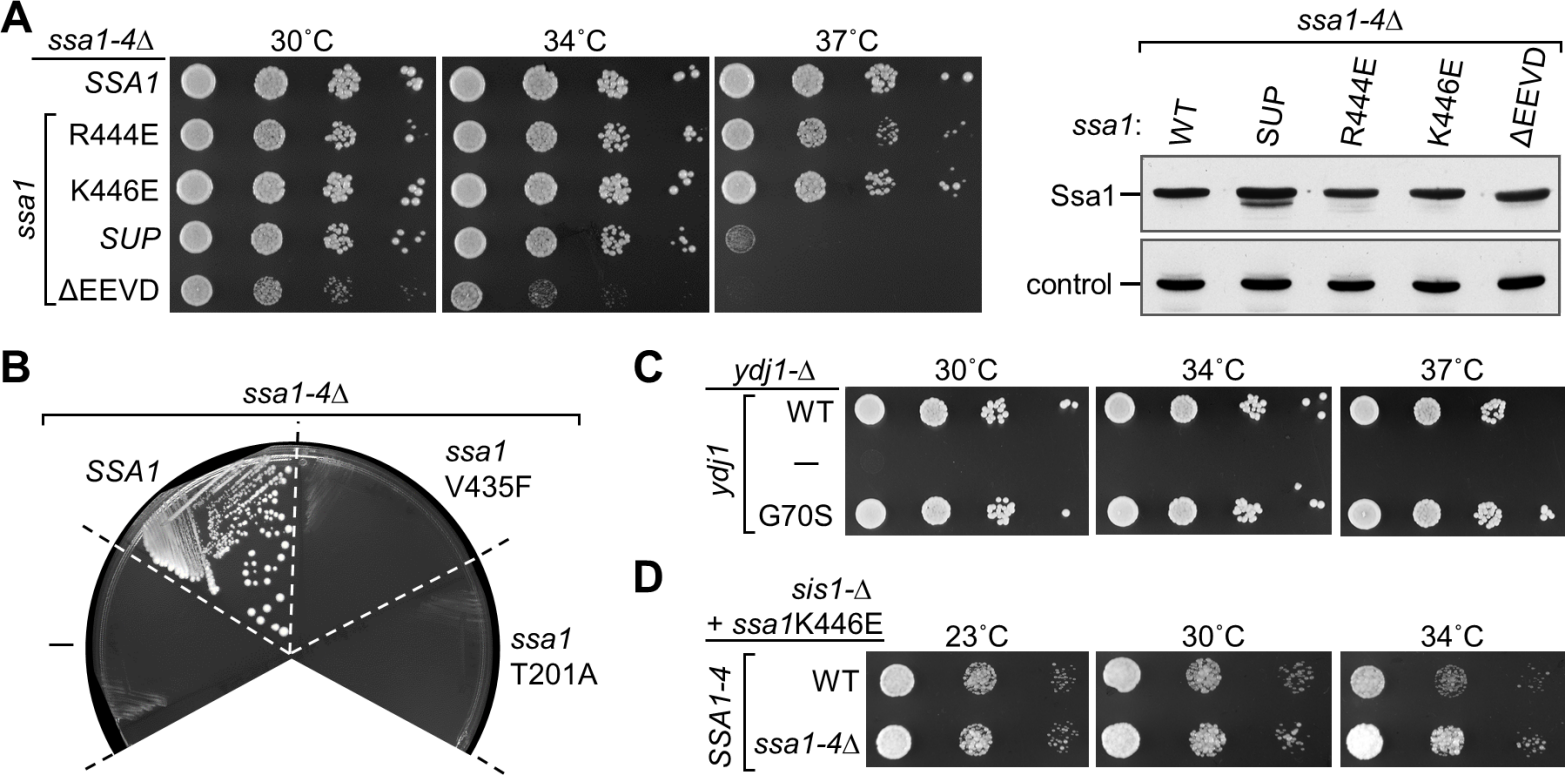
Functionality of Ssa1 suppressor variants in cells lacking WT *SSA* genes. (**A**) (*left*) Ten-fold serial dilutions of a *ssa1-Δ ssa2-Δ ssa3-Δ ssa4-Δ* (*ssa1-4Δ*) strains expressing the indicated *SSA1* gene from a plasmid: WT; original suppressor isolate (*ssa1*_*SUP*_); deletion of codons for four C-terminal residues (ΔEEVD). Plates were incubated at the indicated temperatures for 2 days. (*right*) Cell lysates were subjected to immunoblot analysis to compare Ssa1 levels using antibodies raised against Ssa1 or Tim44 (control). (**B**) *ssa1-Δ ssa2-Δ ssa3-Δ ssa4-Δ* strains harboring two plasmids, one with WT *SSA1* and a *URA3* marker and a second *TRP1*-marked plasmid with no insert (-) or the indicated *SSA1* gene were plated on medium containing 5-FOA. Plates were incubated for 3 days at 30°C. (**C**) Ten-fold serial dilutions of a *ydj1-Δ* strains harboring a plasmid with no insert (-) or a *YDJ1* gene (either WT or with G70S substitution) were plated and incubated at the indicated temperatures for 2 days. (**D**) Ten-fold serial dilutions of *sis1-Δ* cells expressing the *Ssa1*_*K446E*_ from a plasmid, and having either WT or deletions of *SSA1-4* (*ssa1-4-Δ*) on the chromosome. After plating, cells were incubated at the indicated temperatures for either 4 days (23°C) or 3 days (30, 34°C).

The Ssa1 suppressors were selected in a strain having WT *SSA* genes in the chromosome. To assess the ability of Ssa1_R444E_ and Ssa1_K446E_ to support general Hsp70 functionality, we tested the growth of a strain having the four genes that encode *SSA*-type Hsp70s (*SSA1-4*) deleted, and expressing a Ssa1 variant from a plasmid (**Fig 8A**). As a negative control, we tested the Ssa1 variants having the substitutions described above (T201A in the ATPase catalytic site or V435F in the peptide binding cleft). As expected, since Ssa Hsp70s are essential [32], neither negative control supported growth (**Fig 8B**). In addition, we tested the variant lacking the C-terminal EEVD. Cells expressing Ssa1_ΔEEVD_ at levels similar to that of WT Ssa1 were viable, but grew poorly even at 30°C, consistent with a previous report [33] (**Fig 8A**). In contrast, cells expressing the original suppressor isolate, Ssa1_SUP_, Ssa1_R444E_ or Ssa1_K446E_ were viable, growing quite robustly even at 34°C. While cells expressing Ssa1_SUP_ did not form colonies at 37°C, Ssa1_R444E_ or Ssa1_K446E_ allowed single colony formation at this borderline temperature as well. However, growth of *ssa1*_*K446E*_ cells was consistently more robust at 37°C than *ssa1*_*R444E*_.

As the Ssa1 variants are able to support growth of cells having no other Ssa Hsp70s, we also tested the ability of the Ydj1 suppressing variant to support growth of cells lacking WT Ydj1. Ydj1_G70S_, the original isolate, was able to support growth of *ydj1-Δ* cells even at 37°C (**Fig 8C**). Together, these data demonstrate that the suppressor variants are able to carry out essential functions under optimal growth conditions, indicating that the acquisition of the ability to substitute for Sis1 has not eliminated their ability to carry out normal cellular functions.

Lastly, since Ssa1 suppressor variants are able to support growth when they are the only Ssa Hsp70 present in the cell, we asked if the Ssa1_K446E_ variant could support growth when Sis1, as well as WT Ssa protein, are absent. To this end we used plasmid shuffling after construction of a strain having deletions in the chromosome of *SIS1*, *SSA1*, *SSA2*, *SSA3* and *SSA4*. This strain lacking both WT Ssa1 and WT Sis1, but expressing Ssa1_K446E_, grew between 23°C and 34°C, as well as, if not better than cells expressing WT Ssa proteins, in addition to the suppressing variant (**Fig 8D**).

## Discussion

We report here that the lethality caused by the absence of an essential J-protein can be overcome by gain of function variants in either its partner Hsp70 or another J-protein. Although our results do not establish the exact mechanism of suppression, they point to neither client binding nor sub-compartment localization, the two established means of J-protein specialization. Rather, they point to the existence of a third mode, tuning of the Hsp70 substrate interaction cycle, which can be accomplished by changes in or near the J-domain of a J-protein or in Hsp70 itself. We speculate that the suppressor substitutions in Ydj1 and Ssa1 act at the initial part of the cycle, consistent with the well-documented role of J-domains in stimulating Hsp70’s ATPase activity and the diminished ability of peptide substrate to stimulate the ATPase activity of the Ssa1 variants.

The isolated suppressing mutations in *YDJ1* that allowed partial substitution for Sis1 encoded substitutions of either the glycine at the junction point between the J-domain and Gly-rich region or the conserved tyrosine of the J-domain’s helix IV. Overall, our results suggest that these substitutions disturb the local conformation of the broader junction segment, including how helix IV interacts with the rest of the J-domain. Indeed, in the structure presented here, Y66 forms the predominant interactions of Helix IV with the remainder of the J-domain. Moreover, Y66 is conserved, in *E*. *coli* DnaJ and has been proposed to function as a regulator of J-domain function [34–36]. The idea that the interactions of helix IV with the rest of the J-domain are functionally important also explains the rather striking observation that each of the 5 residues we substituted at position 70 led to a degree of suppression. The free-ranging movement of the backbone afforded by the glycine normally present at position 70 may well foster interactions between residues on opposite sides of the junction, helping to stabilize these interactions. In the case of Hsp70, the 444 and 446 suppressing substitutions that render Ssa1 capable of supporting growth of cells lacking Sis1 are predicted from Hsp70 structural information to destabilize the interaction of SBDβ with the lid, SBDα Biochemical analyses of these variants support this idea. Compared to WT Ssa1, both the on- and off-rates of peptide substrate were increased for the variants when bound to ADP. A similar effect on peptide binding was previously observed for a variant of the mammalian Hsp70 of the endoplasmic reticulum lumen, BiP, having an R to E substitution in the residue homologous to R444 [37].

How the suppressor mutations in *YDJ1* and *SSA1* mechanistically affect function remains an open question. In the case of the Ydj1 variants, “loosening” of conformational restraints caused by the substitutions around the J-domain/Gly-region junction may subtly affect how the J-domain interacts with Hsp70, consistent with the slightly reduced ability to stimulate Hsp70’s ATPase activity. Alternatively, functional cooperation between the J-domain and the Gly-rich region may be altered. The Gly-rich region has often been referred to as the “linker region”, as it bridges the J-domain and the substrate-binding β-barrel domain. However, published data point to a significant role of the Gly-rich region [38–40], though its function(s) remains an enigma, and requires further investigation in the future.

In the case of Ssa1, the effect of the suppressing alterations on the interaction between the SBD subdomains is clear. However, the functionally important aspect of this destabilization for suppression is not. On one hand, the decrease in the stability of the interaction of the SBDα lid with the SBDβ points to a decrease in the stability of the interaction with substrate in the ADP-state, as the duration of the trapping of the peptide in the cleft would be reduced. This would manifest itself by an increase in substrate on- and off-rates, particularly in the absence of nucleotide exchange factors. However, based on recent results [13, 41–43], such alterations would also be expected to change the dynamics of interactions between the NBD and SBD domain in the ATP-state (**Fig 7B**). Although the SBDα lid is restrained by its interactions with the NBD when ATP is bound, this is the predominant, not the sole state. It is particularly intriguing that the ATPase activity of 444 and 446 substitution variants was stimulated less effectively than WT Ssa1 by peptide substrate. Despite recent progress in understanding the conformational dynamics of the nucleotide dependent cycle of Hsp70s, little is understood about the role of the lid in stimulation of Hsp70’s ATPase activity by peptide substrate. Our results suggest that lid closure is important for effective stimulation by peptide and perturbation of this step in the Hsp70 cycle supports the suppressing ability of Ssa1 variants. This raises the interesting possibility that the dynamics between lid restraint by the NBD and interaction with SBDβ may play a role in the interdomain allosteric transition by affecting stimulation of ATP hydrolysis by peptide binding.

That we uncovered mutations in both *YDJ1* and *SSA1* that allowed growth of *sis1-Δ* cells, raises the question as to how closely their mechanisms of action are related to each other. It is tempting to speculate that both affect the timing of ATPase stimulation relative to interaction of substrate in the cleft of the SBD. However, before the question of how similar the mechanisms of action of the Ydj1 and Ssa1 suppressing variants are can be answered, more information is needed. For example, better understanding of how J-domains stimulate Hsp70’s ATPase activity, how this stimulation is coordinated with stimulation by substrate binding in the SBDβ cleft and an understanding of SBDα lid dynamics are needed. In addition, for practical reasons, peptide substrates have been used in most of the *in vitro* work with Hsp70s. However, in cells, polypeptides, not peptides, are substrates. In addition, there is a growing body of evidence that partially folded polypeptides are often substrates. In many cases, interaction may occur with a substantially unfolded segment such that lid closure occurs over the bound segment much like that which occurs in model peptide substrate systems. However, in many cases the lid may not close completely upon binding a substrate, as suggested by the analysis of binding of DnaK to its substrate sigma factor 32 [44], as well as recent FRET and single molecule studies with polypeptide substrates [45–47]. This is likely the case for substrates that full-length Sis1 may “deliver” to Hsp70, considering its role in prion propagation [19, 20] and polyglutamine aggregation [48, 49]. One can imagine that the timing of undocking of Hsp70 domains and stimulation of ATPase activity might be more important for larger substrates that are not as easily “trapped” by interaction of the two subdomains of the SBD than for peptide or largely unfolded substrates.

It is important to note that both the Ssa1 and Ydj1 suppressor variants are still able to perform their normal cellular functions, at least to some extent. This supports the idea that the substitutions tune, rather than fundamentally change, the properties of the two proteins. In the case of Ydj1, overexpression of Sis1 can rescue growth defects of *ydj1-Δ* cells [18]. Thus, that a change in Ydj1 able to support viability of *sis1-Δ* can also maintain a level of Ydj1 function may not be as surprising. However, it should be remembered that other chaperones are present in the cell and help partially overcome the absence of WT Ssa or Ydj1. In the case of fungi, these include another class of cytosolic Hsp70s, Ssbs, which, as ribosome-associated Hsp70s, are involved in nascent chain folding [50–52]. Thus, the most critical roles of Ssa Hsp70s that are independent of Sis1 may not be in protein folding per se, but in other processes. Hsp70s are known, however, to be involved in remodeling of protein complexes and intertwining with other cellular quality control pathways [53–56]. In this regard, it is interesting that the deletion of the C-terminal EEVD of Ssa1, while having little effect on the suppressive ability of the variants was very deleterious to the normal functions of WT Ssa1.

Though the results presented here lead us to the view that tuning of the steps of the Hsp70-substrate interaction cycle is an important functional determinant, this does not minimize the relevance of other factors that both distinguish Sis1 and Ydj1 functional abilities and allow them to function in larger chaperone networks [2, 57, 58]. Sis1 and Ydj1 are known to have different substrate binding specificities [59]. In addition, a chimera that contains the J-domain and Gly-rich region of Ydj1 fused to the β-barrel C-terminal segment of Sis1 is sufficient for supporting growth of *sis1-Δ* cells and maintaining some prions [58]. Rather, it is likely that J-proteins have evolved diverse mechanisms, which in combination optimize functionality of each particular J-protein with particular substrates. Consistent with multiple mechanisms, the G70 and Y66 substitutions do not fully compensate for the absence of Sis1. Growth is not as robust as that of a WT strain and it is known that under normal conditions an excess of Sis1 is present, more than is required for optimal growth [60].

In sum, as much of the specificity of Hsp70s’ multiple roles in the cell is due to J-protein co-chaperone function, understanding the mechanistic basis of how such specialization is driven is an important biological question. Next steps entail not only understanding the mechanistic basis behind the suppression observed, but understanding the degree to which tuning of the Hsp70 substrate interaction cycle can be modulated to positively affect biological function.

## Methods

### Strains and media

All yeast strains used in this research were of the W303 genetic background. Most were derived from PJ53, which is isogenic to the W303 background and possesses the following genotype: *trp1-1/trp1-1 ura3-1/ura3-1 leu2-3*,*112/leu2-3*,*112 his3-11*,*15/ his3-11*,*15 ade2-1/ade2-1 can1-100/can1-100 GAL2*^*+*^*/GAL2*^*+*^ *met2- Δ1/met2-Δ1 lys2-Δ/lys2-Δ*) [61]. For testing the function of Sis1 suppressors in the absence of WT *SIS1*, WY26 (α *trp1-1 ura3-1 leu2-3*,*112 his3-11*,*15 ade2-1 can1-100 GAL2*^*+*^ *met2-Δ1 lys2-Δ2 sis1*::*LEU2* (which we call *sis1-Δ* throughout the text) carrying YCP50-*SIS1* [23] was transformed with plasmids encoding the suppressors. Colonies having lost the plasmid WT *SIS1* (YCP50-*SIS1*) were selected for on complete minimal media plates containing 5-fluoroorotic acid (5-FOA) (Toronto Research Chemicals Inc., Canada) [62]. WY26 is [*RNQ*^*+*^] [*psi*^*-*^]. For analysis of [*PSI*^*+*^], strains containing either [*PSI*^*+*^]^*Sc4*^ or [*PSI*^+^]^*Sc37*^ were deleted for *SIS1* as previously described [19]. The [*PSI*^*+*^] strains carry an *ade1-14* allele rather than an *ade2-1* allele which provides a plate color assay to assess [*PSI*^*+*^] maintenance.

To purify Ssa1 proteins from yeast, a quadruple *SSA* knockout strain was constructed. The ORFs for *SSA1* and *SSA2* were replaced with *nat1MX4* and *kanMX4*, respectively. First, PCR fragments containing *ssa1*::*kanMX4* and *ssa2*::*kanMX4* were amplified from the genome knockout collection (Open Biosystems, Huntsville, AL) with primers designed to anneal to the 5’ and 3’ UTR regions of these genes using Phusion polymerase (New England BioLabs, Ipswich, MA) and used to transform haploid strains of the opposite mating types. The *kanMX4* marker of *ssa1*::kanMX4 was exchanged with the *nat1MX4* marker by transforming the strain with an *Eco*RI-*Not*I fragment from pAG36 [63]. The genes for *SSA3* (+91 to +1743) and *SSA4* (-95 to +2075) were replaced with the *LYS2* and *ADE2* genes, respectively, in the *ssa2*::*kanMX4* haploid strain. The resulting strain (BAS41) was crossed to the *ssa1*::*nat1MX4* strain and the isolated diploid was transformed with p416-TEF-*SSA1*, sporulated, and dissected to obtain the haploid quadruple knockout strain, BAS42. Strains were verified by PCR of yeast genomic DNA, isolated using the MasterPure Yeast DNA Purification Kit manufactured by Epicentre (Madison, WI), using primers specific to the integrated DNA.

In order to test the ability of the *sis1-Δ* suppressor mutation in *SSA1* to functionally replace both Sis1 and Ssa1 proteins, WY26 was crossed with BAS42. The diploid was plated on 5-FOA to isolate a strain without the *URA3* plasmids. This diploid was transformed with YCP50-*Sis1* and p414-TEF-*SSA1*_*K446E*_ (see below), sporulated, and dissected. A haploid containing all five deletions and both plasmids was placed on 5-FOA to produce the following strain, *sis1-Δ ssa1-Δ ssa2-Δ ssa3-Δ ssa4-Δ* + p414-TEF*-SSA1*_*K446E*_.

Yeast strains were grown in YPD (1% yeast extract, 2% peptone (Difco laboratories, Detroit MI), 2% dextrose), YPAD (YPD plus 40 mg/L adenine) or selective minimal media (0.67% yeast nitrogen base without amino acids (US Biological, Marblehead, MA), 2% dextrose), supplemented with required amino acids [64]. Yeast were transformed using a previously developed protocol [65]. To analyze cell growth, 10-fold serial dilutions of cells were spotted onto selective minimal media and grown for the recorded number of days at the indicated temperatures. Representative examples are shown for both serial dilution and platings on 5-FOA, with all experiments repeated a minimum of three times.

The *E*. *coli* strain DH5α was used for general cloning procedures and Rosetta 2 (DE3) pLysS *E*. *coli* cells (Novagen, Madison, WI) were used for purification of Ydj1 WT and variant proteins. *E*. *coli* strains were grown either in Luria broth or in M9 media, for ^15^N or ^15^N ^13^C labeling [66], along with the addition of necessary antibiotics for plasmid maintenance.

### Plasmid constructions

Plasmid pYW65 (pRS314-*SIS1*) has been described previously [23]. pYW15 (pRS314-*YDJ1*) was constructed by isolating a 4.3 kb *Nhe*I fragment containing *YDJ1* from a genomic library clone and ligating with pRS314 digested with *Spe*I. pMAL-His-TEV-*YDJ1*, a plasmid expressing Ydj1 for purification, was previously described [67]. Regions encoding Ydj1_1-70_ (J-domain) and Ydj1_1-109_ (J-domain and Gly-rich region) were cloned into pMAL-His-TEV as PCR products with a 5’ *Bam*HI site and a 3’ *Sal*I site in order to purify proteins for NMR analysis and into p414-TEF [68] to test *in vivo* function. pRS314-TEF-His-*SSA1* is a plasmid for purifying Ssa1 from yeast and was created by moving a *Sac*I/*Not*I fragment (3.6 kb) from p416-TEF-His-*SSA1* [28] into pRS314 similarly digested. The open reading frames for *YDJ1* and *SSA1* were each cloned by PCR amplification from genomic DNA with primers introducing a 5’ *Bam*HI site and a 3’ *Xho*I site. The restriction digested PCR products were ligated into p414-TEF similarly digested. All site-directed mutations made in these plasmids were obtained using the QuikChange protocol (Stratagene, La Jolla, CA). All plasmids were sequenced at the University of Wisconsin Biotechnology facility for verification.

### Suppressor selection and library constructions

Spontaneous suppressors were obtained by streaking WY26 transformed with pRS314 from a Trp omission plate to a 5-FOA plate. After 3 days of growth at 30°C and an additional 3 days growth at room temperature, colonies that developed were tested for the presence of Sis1 by immunoblot analysis of lysates separated by SDS-PAGE and transferred to nitrocellulose using anti-Sis1 antibodies [23]. The healthiest suppressor, which did express detectable levels of Sis1, was crossed back to a WT haploid. It was determined through sporulation and tetrad dissection that the suppressor mutation(s) segregated with the *sis1-Δ* allele based on the fact that from 103 complete tetrads, 59 were tetratype, 41 were parental ditype, and only 3 were non-parental ditype. Upon examination of chromosome XIV, it was observed that *YDJ1* lies 111.4 kb away from *SIS1*. *YDJ1* from the *sis1-Δ* suppressor strain was cloned as described above and found to carry a single nucleotide change from G to A at position +208, resulting in a codon change from glycine (GGT) to serine (AGT).

To obtain *sis1-Δ* suppressor mutations in either *YDJ1* or *SSA1*, plasmid libraries containing randomly mutagenized DNA through error prone PCR amplification were generated. A truncated gene for *YDJ1* was amplified using a 5’ primer with a *Bam*HI site beginning at the initiating methionine and a 3’ primer with a *Xho*I site and stop codon annealing after codon 134. The digested product was ligated to p414-TEF similarly digested and transformed into *E*. *coli*, resulting in ~14,000 independent transformants. The entire ORF of *SSA1* was amplified using a 5’ primer with a *Bam*HI site adjacent to the initiating methionine and a 3’ primer with a *Xho*I site annealing after the stop codon. The digested product was ligated to p414-TEF similarly digested and transformed into *E*. *coli*, resulting in ~13,000 independent transformants.

Library transformants of *sis1-Δ* were plated on ten Trp omission plates and grown for three days at 30°C yielding ~1000 colonies per plate. The cells from each plate were resuspended in 5 ml of water and 100 μl of each suspension was plated onto 5-FOA plates. Plates were grown at 30°C and examined daily for the appearance of faster growing colonies. Lysates made from suppressor candidates were tested for the presence of Sis1 by immunoblot analysis using anti-Sis1 antibodies. Plasmid DNA was rescued from suppressor candidates which did not show detectable levels of Sis1. *sis1-Δ* was transformed with the rescued plasmids and suppression of *sis1-Δ* lethality was tested by observing growth on 5-FOA plates. The initial *ssa1*_*SUP*_ contained mutations resulting in 7 amino acid substitutions (E104G, K136E, I213L, Y368H, R444G, S479T and K592M). Each substitution was created individually using the QuikChange protocol and tested *in vivo* by plasmid shuffling in *sis1-Δ*. Only R444G allowed formation of colonies on 5-FOA plates.

### Prion maintenance assays

To test the ability of Ydj1 and Ssa1 variants to replace Sis1’s role in the maintenance of prions [*RNQ*^+^] and [*PSI*^+^], [*PRION*^+^] *sis1-Δ* cells carrying YCP50-*SIS1* were transformed with pRS414 encoding the Ydj1 or Ssa1 variants. All incubations were carried out at 30°C, unless stated otherwise. Transformants were selected on minimal media plates lacking tryptophan and subsequently transferred to 5-FOA plates to counter-select against the *URA3*-marked plasmid expressing WT Sis1. Uracil auxotrophy and tryptophan prototrophy were confirmed. For the [*RNQ*^+^] maintenance experiments, post-5-FOA cells were grown on selective minimal media plates for 2 days before preparation of lysates. The slower-growing [*PSI*^+^] cells were transferred to and re-plated twice on YPAD to provide more time for potential prion-loss. Cells were grown on YPD plates for 4–5 days at 23°C, and the color phenotype was examined for the presence or absence of [*PSI*^+^].

The presence of detergent resistant aggregates was determined using SDD-AGE. Yeast cells were grown overnight at 30°C in selective minimal media and YPAD media for [*RNQ*^+^] and [*PSI*^+^] experiments, respectively. 8–10 OD_600_ units of log phase cells (OD_600_ of 0.8–1.0) were pelleted and washed with water. Cell lysates were made according to a previously published technique [69]. Protein concentrations of the lysates were determined using the Bio-Rad protein assay reagent (Bio-Rad, Hercules, CA). Equivalent protein samples were separated using SDD-AGE followed by transfer to nitrocellulose and immunoblot analysis [19].

### Protein purification

His-Ssa WT and variant proteins were purified as previously described [28] with some modifications. The His-tagged proteins were expressed in BAS42 after plasmid shuffle of p416-TEF-*SSA1* with the purification vector, pRS314-TEF-His-*SSA1*. Two liters of cells were grown overnight in YPD at 30°C to an OD_600_ of 3–4. Pelleted cells were resuspended in 80 ml of buffer A (5 mM imidazole, 0.5 M NaCl, 20 mM Tris, pH 8.0, 5% glycerol, and protease inhibitor tablets (cOmplete Mini, EDTA-free from Roche Diagnostics Indianapolis, IN)) and lysed by passage through a TS2 cell disrupter manufactured by Constant Systems, Ltd (Northants, UK). The cleared lysate was batch incubated with 5 ml of His-Bind Resin (Novagen, Madison, WI) for 1 hr at 4°C. The resin was collected by centrifugation and transferred to a column where it was washed with 100 ml buffer A prior to elution of the protein with 25 ml of buffer A containing 300 mM imidazole using gravity flow. Fractions containing Ssa1 protein were pooled and diluted fivefold in ATP-agarose binding buffer B (20 mM Tris, pH 7.5, 50 mM NaCl, 10 mM MgCl2, 5 mM 2-mercaptoethanol, 5%) and incubated with 3.0 ml ATP-agarose beads (Sigma Chemical Co.) in batch for 2 hrs at 4°C. The beads were collected, transferred to a column, and washed sequentially with 30 ml buffer B, 30 ml of the buffer B containing 500 mM NaCl, and finally with 10 ml of buffer B. Ssa1 was eluted with 20 ml of buffer B containing 5 mM ATP. Fractions containing Ssa1 were pooled, concentrated and passed over a PD10 column (GE Healthcare, UK) equilibrated with the final storage buffer (20 mM Tris, pH 7.5, 20 mM NaCl, 10 mM MgCl2, 5 mM 2-mercapthoethanol, 10% glycerol). Aliquots of protein were flash frozen in liquid N_2_ and stored at -80°C.

WT Ydj1 and variants were purified as previously described with some modifications [67]. Cells pelleted from a liter of media were resuspended in 20 ml of buffer C (20 mM Tris, pH 7.5, 500 mM NaCl, 20 mM imidiazole, pH 7.5, with a protease inhibitor tablet) and lysed by two passages through a French pressure cell. After washing and elution from the His-Bind Resin, the fractions containing the MBP-Ydj1 fusion protein were pooled, diluted 50% with dialysis buffer (20 mM Tris, pH 7.5, 150 mM NaCl, 1 mM DTT), and placed into a dialysis bag with TEV protease. Equilibration and proteolysis occurred for at least 18 hours in 4 liters of dialysis buffer. The purification continued as previously described with the addition of a gel filtration purification step using a HiLoad 16/60 Superdex 75 or HiPrep 16/60 Sephacryl S-200 HR prep grade columns from GE Healthcare UK (Buckinghamshire, UK) equilibrated with dialysis buffer. Protein samples were concentrated using Amico^®^ Ultra centrifugal filter devices (Merck EMD Millipore Corporation, Billerica, MA) following manufacturer instructions. Protein concentrations were determined using the Bio-Rad protein assay reagent (Bio-Rad, Hercules, CA) and purity determined by running SDS-PAGE and staining with Coomassie Blue. All samples prepared for NMR contained more than 300 μM uniformly ^13^C and/or ^15^N labeled protein in buffer (20 mM Tris, pH 7.5, 150 mM NaCl, 5 mM DTT) with protease inhibitor tablets, 0.02% NaN_3_ and 7% ^2^H_2_O added.

### Immunoblot analyses and antibodies

All immunoblot analyses were carried out using the Enhanced Chemi-luminescence system (GE Healthcare, UK, Buckinghamshire, UK) according to the manufacturer’s suggestion, by using polyclonal antibodies specific for Sis1 [67], Ydj1 [23], Ssc1 [70], Tim44 [70], Ssa1 [71], Rnq1 [22] and Sup35 [19].

To purify antibodies specific to the J domain of Ydj1 (1–70), anti-Ydj1 antisera was incubated with nitrocellulose strips containing purified Ydj1_1-70_. The strips were thoroughly washed and the antibody eluted using 100 mM Glycine pH 2.5. The antibody was neutralized with 1M Tris-HCl pH8.5, brought to 150 mM NaCl, and NaAzide was added to 0.1%.

### Anisotropy assays

For equilibrium measurements, fluorescein-labeled P5 (F-P5, Flc-CALLLSAPRR, Sigma Chemical, St. Louis, MO) at 10 nM final concentration was combined with Ssa1 at various concentrations (2-fold increments from 2.4 nM to 5 μM) in buffer A (25 mM Hepes-KOH pH 7.5, 100 mM KCl, 11 mM MgOAc) containing a final concentration of 1 mM ADP, 4% (v/v) glycerol, and 0.005% (v/v) NP-40. After 24 hr incubation at room temperature (23°C), polarization was measured using a Beacon Fluorescence Polarization System (PanVera Corporation, Madison, WI). Control samples lacking Ssa1 were used for background subtraction. Data points were plotted using SigmaPlot 11 (Systat Software, Inc., San Jose, CA) and K_d_ values obtained using a one site saturation formula for ligand binding.

For kinetic analysis of binding, half reactions were prepared in buffer A containing either Ssa1 and ADP or F-P5. Final concentration of components was 5 μM Ssa1, 1 mM ADP, 10 nM F-P5, 4% glycerol, and 0.005% NP-40. A control reaction lacking Ssa1 was used for initial measurement. Reactions were initiated by the addition of F-P5 solution to a tube containing Ssa1 and ADP. After rapid mixing, polarization was measured at intervals of 10 seconds. Polarization values were used to calculate percent of maximal binding after subtraction of background (t = 0 value). Maximum binding is defined as the equilibrium polarization value obtained at 5 μM Ssa1. The resulting percent binding values were plotted using SigmaPlot 11 (Systat Software, Inc., San Jose, CA), with curves fitted using a 2 parameter hyperbola equation.

For kinetic analysis of Ssa1/F-P5 dissociation, samples containing 5 μM Ssa1, 1 mM ADP, 10 nM F-P5, 4% glycerol, and 0.005% NP-40 in buffer A were allowed to reach equilibrium as described above. Reactions were initiated by the addition of 2 μl of a solution of peptide P5 (CALLLSAPRR, Sigma Chemical, St. Louis, MO) in buffer A + 0.01% NP-40 to achieve a final concentration of 40 μM P5. After rapid mixing of the reaction, polarization values were determined at 10 sec intervals. The percent complex remaining was calculated by defining the polarization value at t = 0 as 100% binding. Samples lacking Ssa1 were used for background subtraction. The resulting values were plotted and curves fitted using a 3 parameter hyperbolic decay equation.

### ATPase assay

Single turnover ATPase assays were performed essentially as described previously [67]. Briefly, 30 μg of Ssa1 was combined with approximately 10 μCi α^32^P-ATP in buffer A containing ATP at 10 μM final concentration. Ssa1 with bound ATP was separated from free nucleotide using a spin column (illustra MicroSpin G-25, GE Healthcare, Chicago, IL) and brought to 10% glycerol (v/v) before preparing aliquots and freezing in liquid N2. Assays were performed at 27°C in a volume of 50 μl. Reactions were initiated by the addition of pre-formed α^32^P-ATP/Ssa1 complex to a pre-equilibrated reaction mixture. At defined time points, 3 μl samples were combined with 1 μl stop solution (2 M LiCl, 16% [w/v] formic acid, 10 mM ATP). For each time point, the ADP/ATP ratio was determined by thin layer chromatography (Polygram CEL 300 PEI, Macherey-Nagel, Duren, Germany), followed by phosphorimaging using a Typhoon FLA-9000 Imager (GE Healthcare, Chicago, IL). The percent hydrolysis over time was used to calculate the hydrolysis rate. Data were plotted using SigmaPlot 11 (Systat Software, Inc., San Jose, CA).

### NMR Data collection and processing

NMR spectra were collected using Varian VNMRS and Bruker Avance spectrometers operating at 600, 800 or 900 MHz, and equipped with cryogenic triple-resonance probes. Protein samples used for NMR experiments were labeled uniformly with ^15^N or ^15^N and ^13^C. NMRPipe software [72] was used to process NMR spectra, and NMRFAM-Sparky software [73] was used for spectral analysis. For backbone resonance assignments, data were acquired from the following two-dimensional (2D) and three-dimensional (3D) NMR experiments: 2D ^1^H, ^15^N HSQC, 3D HNCO, 3D HNCACB, 3D CBCA(CO)NH, 3D HNCA and 3D HN(CO)CA. For Ydj1_70_, Ydj1_70_WT and Ydj1_109_G70N, NMR data were collected at 6°C, 25°C and 10°C, respectively. Data from additional NMR experiments, acquired at 6°C, were used in determining the side chain resonances assignments of Ydj1_70_: 2D ^1^H, ^13^C^aliphatic^/^13^C^aromatic^ HSQC, 3D HBHA(CO)NH, 3D C(CO)NH, 3D H(CCO)NH, 3D H(C)CH-TOCSY. Data for structure determinations were acquired from the following NMR experiments: 3D NOESY ^15^N-HSQC, 3D NOESY ^13^C^aliphatic^-HSQC and 3D NOESY ^13^C^aromatic^-HSQC spectra with 100ms mixing time. All 3D spectra were recorded using non-uniform sampling (NUS) with a sampling rate of 50%. The software package SMILE[74], available in NMRPipe [72], was used to reconstruct spectra from the NUS data. ^15^N-^1^H residual dipolar coupling (RDC) measurements provided information about the orientation of internuclear vectors of interest relative to the molecular frame under minute alignment in the external magnetic field. A sample of Ydj1_70_ in a neutral acrylamide gel was partially aligned by stretching the gel from 5.4 to 4.2 mm [75]. An IPAP-HSQC experiment [76], carried out at 10°C, was used to collect the coupling data for RDC measurements.

### NMR analysis

#### Resonance assignments

Signals for sequence-specific backbone resonance assignments were detected by APES [77] and assigned by PINE algorithm [78], and followed with subsequent manual verification by PINE-SPARKY [79]. Due to TEV cleavage site introduced into purification constructs, GEFGS is present at the N-terminus, followed by the methionine at position 1 of the native Ydj1 protein sequence. Backbone resonances of D36 and the N-terminal GE residues could not be assigned. For Ydj1_70_, backbone resonances of all remaining non-proline residues were identified. Predict-and-confirm method was used to obtain nearly complete side chain resonance assignments [80]. To perform backbone resonance assignments for both Ydj1_109_WT and Ydj1_109_G70N, an additional pair of 3D HNCA and 3D HN(CO)CA spectra were used to resolve resonance overlap in the glycine rich region. Specifically, very high resolution of these spectra was achieved by using selective ^13^C pulses and a very narrow spectral window focused on the Cα region of glycine residues only. This approach allowed assignment of backbone resonances for all remaining non-proline residues of Ydj1_109_WT. For Ydj1_109_G70N, of the 104 non-proline residues identified for Ydj1_109_WT, resonances for 88 residues were assigned; 16, including those from residues T5-D9 and L57-Q68, could not be identified. In addition, the G70N substitution led to replacement of the G70 signal by a new signal from N70.

#### Chemical shift analysis

The chemical shift perturbation (CSP) analysis was performed by comparing the positions of signals in 2D ^1^H, ^15^N HSQC spectra of Ydj1_109_WT and Ydj1_109_G70N, recorded at 10°C. Chemical shift differences were determined in the ^1^H (δ_H_) and ^15^N (δ_N_) dimensions, and these were converted to a consensus chemical shift difference (_Δ_δ_HN)_ by using the equation: _Δ_δ_HN_ = [(_Δ_δ_H_)^2^ + (_Δ_δ_N_/5)^2^]^½^.

#### NMR structure determination

The solution NMR structure of Ydj1_70_ was determined using Xplor-NIH based calculations from the PONDEROSA-C/S package [73, 81]. To obtain distance and angle constraints, we chose PONDEROSA-X refinement that runs AUDANA algorithm [82] with TALOS-N optimization [83]. Resonance assignments, 3D NOESY and RDC data were used as inputs. After a few rounds of constraint validations and iterative structure calculation with Constraints Only-X calculations, final calculations were run with Final step with explicit water refinement option that refines the 20 most energetically stable structures out of 100 calculated structures in the water box. We used PSVS suites to validate the calculated structures[84–87].

#### Miscellaneous

All chemicals used in this study were purchased from Sigma-Aldrich (St. Louis, MO) unless noted otherwise. Restriction enzymes were purchased from New England BioLabs (Ipswich, MA). The ^13^C labeled glucose and ^15^N labeled ammonium chloride were purchased from Cambridge Isotope Laboratories, Inc. (Andover, MA). Homology modeling of Ssa1 structures in ATP and ADP state were prepared using SWISS-MODEL [88] and DnaK structures (PDB: 4NJ4 and 2KHO, respectively) as templates. Structural images were prepared with the PyMOL Molecular Graphics System, Version 1.5 Schrödinger, LLC.

### Accession numbers

Coordinates and related data have been deposited at the Research Collaboratory for Structural Bioinformatics Protein Data Bank under accession number 5VSO and the NMR data at Biological Magnetic Resonance Bank under accession number 30293.

## Supporting information

S1 Table NMR and refinement statistics for Ydj1 J-domain structure. Statistics from the validation reports for the NMR structures of Ydj1 determined by PONDEROSA-C/S with final step calculation with subsequent explicit water refinement option that selects best 20 of 100 structures based on lowest pseudo-potential energy criteria. PROCHECK and MOLPROBITY validation reports were generated from PSVS web server.
(TIF)

S1 Fig Analysis of prion aggregates in [*PSI*^*+*^]^Sc37^ and [*RNQ*^*+*^] *sis1-Δ* strains carrying *ydj1* or *ssa1* mutations. (**A**) Cell lysates, made from WY26 strains in which the *URA3* marked plasmid carrying WT *SIS1* was shuffled with plasmids carrying the indicated *ydj1* or *ssa1* mutant gene, were separated using SDD-AGE, transferred to nitrocellulose and immunoblotted with Rnq1-specific antibodies. A lysate from WY26 which was cured of its prion ([*rnq*^*-*^]) was run as a negative control. (**B**) Cell lysates made from [*PSI*^*+*^]^Sc37^ *sis1-Δ* strains carrying either WT *SIS1* ([*PSI*^*+*^]) or the indicated *ssa1* mutations were separated using SDD-AGE, transferred to nitrocellulose and immunoblotted with Sup35-specific antibodies. Lysate of the parental strain cured of the ([*psi*^*-*^]) was run as a negative control.
(TIF)

S2 Fig Interaction of Ssa1 (WT and variants) with peptide P5. Equilibrium binding of F-P5 over a range of Ssa1 concentrations. Indicated concentrations of Ssa1 were combined with F-P5 (10 nM final concentration) in the presence of 1 mM ADP. Reactions were incubated at room temperature until equilibrium was reached. Symbols represent the mean of 4 independent experiments. Error bars indicate the standard deviation of the mean for each set of values. Curve fitting is based on single site ligand binding.
(TIF)
