# Supplementary figures and images for: Broadening the functionality of a J-protein/Hsp70 molecular chaperone system
Source: PLoS Genet. 2017 Oct 30;13(10):e1007084. doi: 10.1371/journal.pgen.1007084 (PMC5679652; doi:10.1371/journal.pgen.1007084)

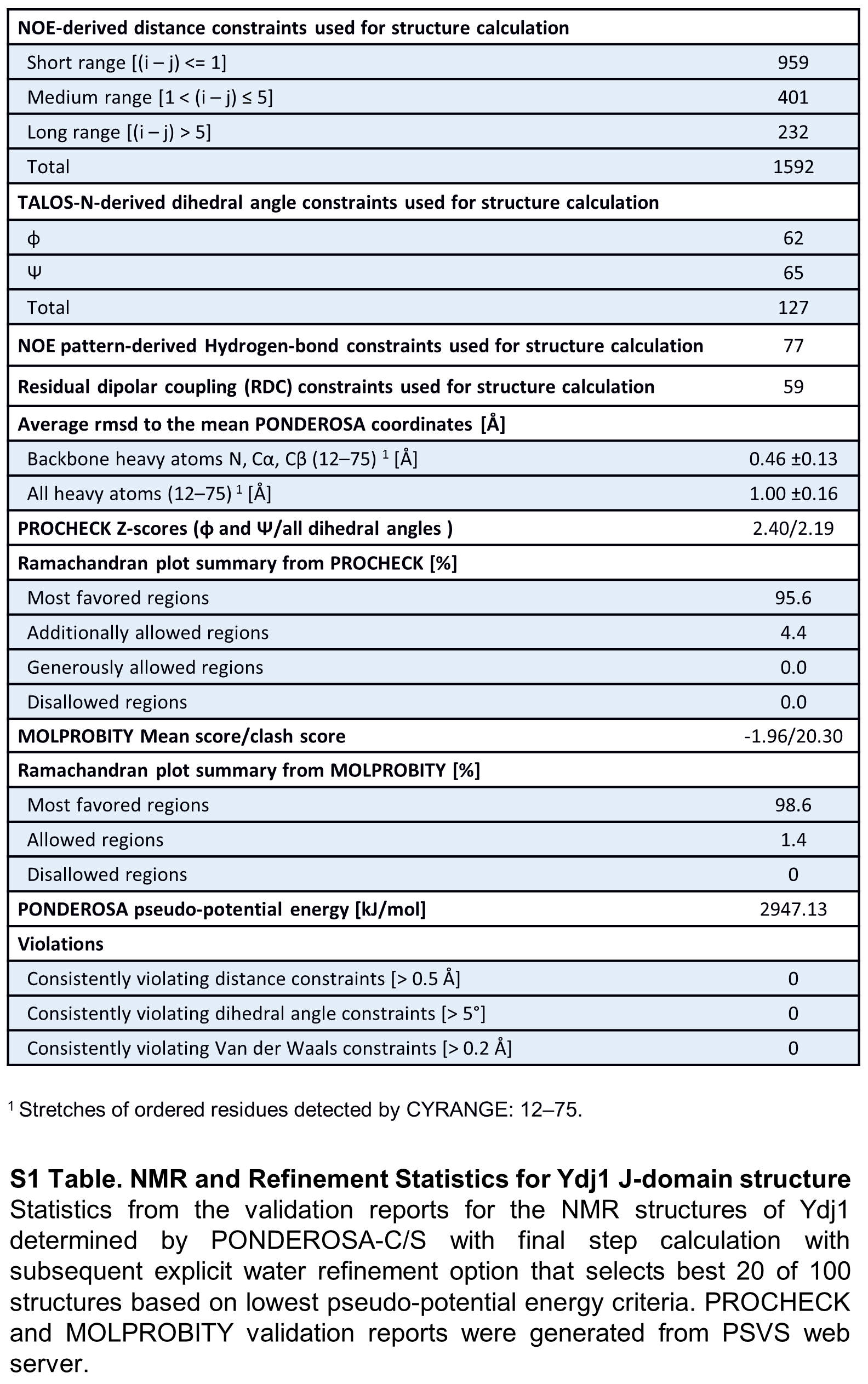

Supplement: S1 Table — Statistics from the validation reports for the NMR structures of Ydj1 determined by PONDEROSA-C/S with final step calculation with subsequent explicit water refinement option that selects best 20 of 100 structures based on lowest pseudo-potential energy criteria. PROCHECK and MOLPROBITY validation reports were generated from PSVS web server. (TIF) [file pgen.1007084.s001.tif]

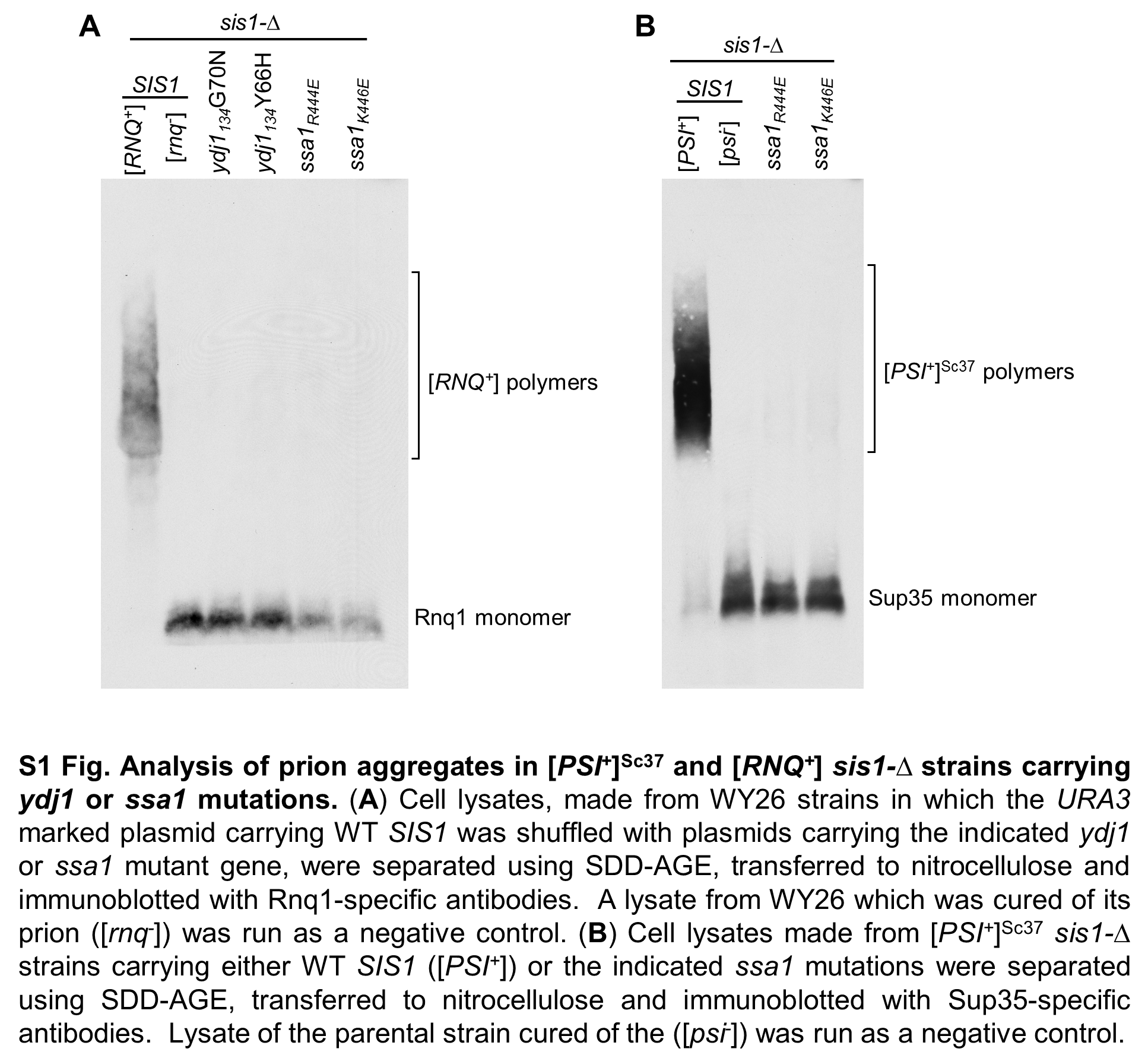

Supplement: S1 Fig — (A) Cell lysates, made from WY26 strains in which the URA3 marked plasmid carrying WT SIS1 was shuffled with plasmids carrying the indicated ydj1 or ssa1 mutant gene, were separated using SDD-AGE, transferred to nitrocellulose and immunoblotted with Rnq1-specific antibodies. A lysate from WY26 which was cured of its prion ([rnq-]) was run as a negative control. (B) Cell lysates made from [PSI+]Sc37 sis1-Δ strains carrying either WT SIS1 ([PSI+]) or the indicated ssa1 mutations were separated using SDD-AGE, transferred to nitrocellulose and immunoblotted with Sup35-specific antibodies. Lysate of the parental strain cured of the ([psi-]) was run as a negative control. (TIF) [file pgen.1007084.s002.tif]

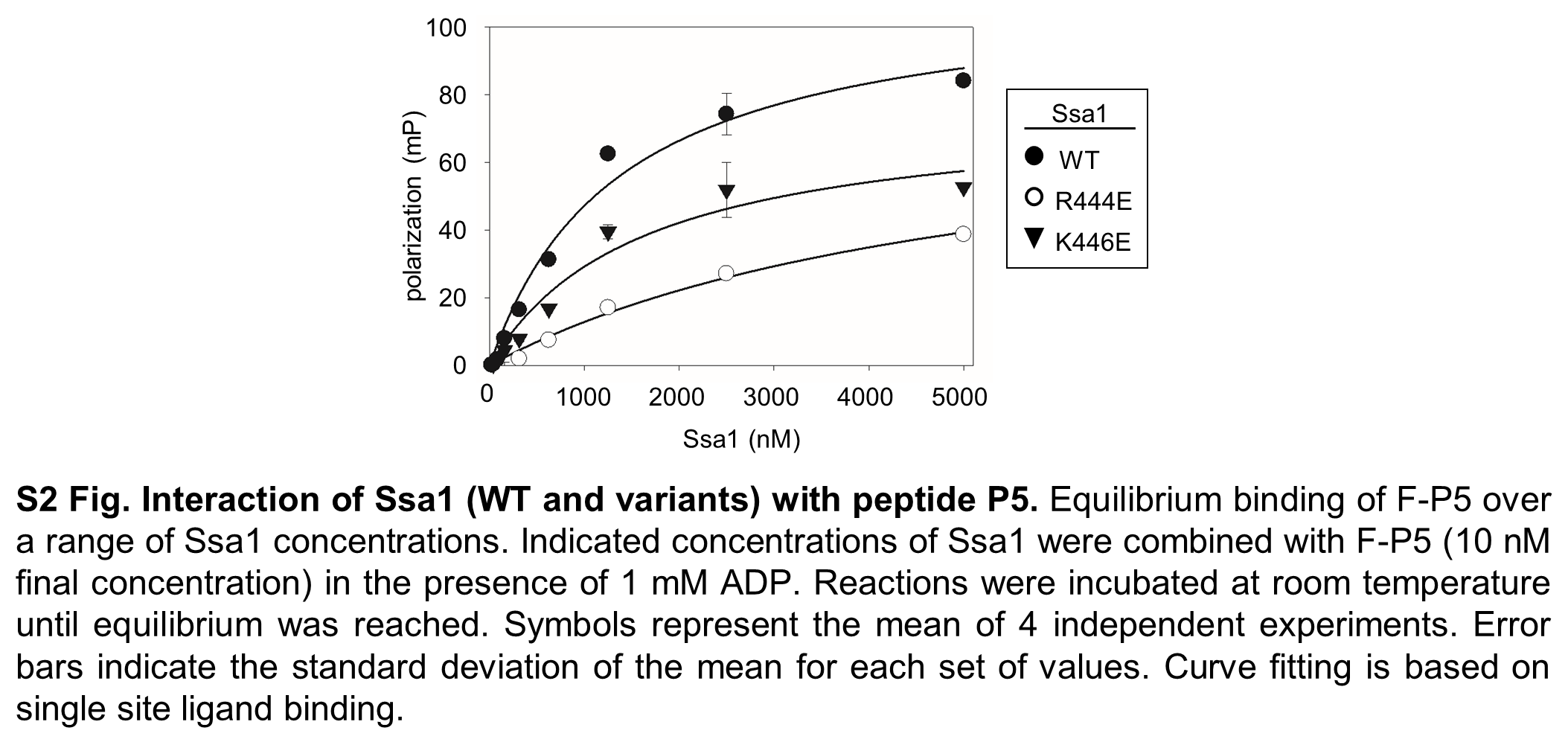

Supplement: S2 Fig — Equilibrium binding of F-P5 over a range of Ssa1 concentrations. Indicated concentrations of Ssa1 were combined with F-P5 (10 nM final concentration) in the presence of 1 mM ADP. Reactions were incubated at room temperature until equilibrium was reached. Symbols represent the mean of 4 independent experiments. Error bars indicate the standard deviation of the mean for each set of values. Curve fitting is based on single site ligand binding. (TIF) [file pgen.1007084.s003.tif]
